# Metabolomics and Transcriptomics Integration of Early Response of *Populus tomentosa* to Reduced Nitrogen Availability

**DOI:** 10.3389/fpls.2021.769748

**Published:** 2021-12-08

**Authors:** Min Chen, Yiyi Yin, Lichun Zhang, Xiaoqian Yang, Tiantian Fu, Xiaowei Huo, Yanwei Wang

**Affiliations:** ^1^National Engineering Laboratory for Tree Breeding, Key Laboratory of Genetics and Breeding in Forest Trees and Ornamental Plants, Ministry of Education, The Tree and Ornamental Plant Breeding and Biotechnology Laboratory of National Forestry and Grassland Administration, College of Biological Sciences and Biotechnology, Beijing Advanced Innovation Center for Tree Breeding by Molecular Design, Beijing Forestry University, Beijing, China; ^2^School of Life Sciences, Tsinghua University, Beijing, China

**Keywords:** metabolome, transcriptome, poplar, nitrogen deficiency, carbon

## Abstract

Nitrogen (N) is one of the most crucial elements for plant growth and development. However, little is known about the metabolic regulation of trees under conditions of N deficiency. In this investigation, gas chromatography-mass spectrometry (GC-MS) was used to determine global changes in metabolites and regulatory pathways in *Populus tomentosa*. Thirty metabolites were found to be changed significantly under conditions of low-N stress. N deficiency resulted in increased levels of carbohydrates and decreases in amino acids and some alcohols, as well as some secondary metabolites. Furthermore, an RNA-sequencing (RNA-Seq) analysis was performed to characterize the transcriptomic profiles, and 1,662 differentially expressed genes were identified in *P. tomentosa*. Intriguingly, four pathways related to carbohydrate metabolism were enriched. Genes involved in the gibberellic acid and indole-3-acetic acid pathways were found to be responsive to low-N stress, and the contents of hormones were then validated by high-performance liquid chromatography/electrospray ionization tandem mass spectrometry (HPLC-ESI-MS/MS). Coordinated metabolomics and transcriptomics analysis revealed a pattern of co-expression of five pairs of metabolites and unigenes. Overall, our investigation showed that metabolism directly related to N deficiency was depressed, while some components of energy metabolism were increased. These observations provided insights into the metabolic and molecular mechanisms underlying the interactions of N and carbon in poplar.

## Introduction

Plant growth is perturbed by various biotic and abiotic stresses (Rejeb et al., 2014). Among abiotic stresses, nitrogen (N) stress has a major effect on plant physiological activity. Many biological molecules, including nucleic acids, amino acids, proteins, chlorophyll, lipids, and a variety of other metabolites, contain N, which is required for their synthesis (Kusano et al., 2011). N is actively transported or taken up by the plant root system. Organisms utilize N from three sources. The first source is N_2_ from the air, which can be assimilated by leguminous rhizobia (Zahran, 1999), and the second is organic N in the soil, which can be taken up by plants in specific environments (Jones et al., 2005). However, the main sources of N, which are taken up by most higher plants *via* transporters, are ammonium (NH_4_^+^) and nitrate (NO_3_^–^) from the soil (Jackson et al., 2008). The absorption and utilization of N (NH_4_^+^ and NO_3_^–^) are highly regulated in plants (Patterson et al., 2010). A wide range of physiological activities of plants is disrupted by N deficiency, including photosynthesis, signal transduction, and the synthesis of phospholipids, endogenous hormones, and many secondary metabolites (Shao et al., 2020; de Bang et al., 2021; Mu and Chen, 2021).

Nitrogen (N) deficiency usually reduces amino acid synthesis (Albinsky et al., 2010), while metabolite profiling analyses of *Arabidopsis* and maize have shown that growth under low-N conditions causes increases in the levels of many amino acids (North et al., 2009; Broyart et al., 2010; Trachsel et al., 2013). This may be due to varying experimental conditions. For example, gamma-aminobutyric acid (GABA) was unaltered under conditions of nitrate deficiency in high-light conditions but was induced in low-light conditions in tomatoes (Urbanczyk-Wochniak and Fernie, 2005). Furthermore, N deficiency was shown to affect the biosynthesis of some carbohydrates, as the fundamental processes of carbon (C) and N metabolism are tightly coordinated (Cao et al., 2019). The C skeleton and energy provided by carbohydrates are required for photosynthesis and N uptake (Gutieìrrez et al., 2005; Zheng, 2009). It has been demonstrated that N deficiency suppresses the levels of carbohydrate and major soluble sugars, but stimulates the accumulation of starch (Scheible et al., 1997). A wide range of genes is involved in the low-N stress response in plants (Shi et al., 2016). Some N assimilation process-related genes, especially those involved in the ornithine urea cycle (OUC) and tricarboxylic acid (TCA) cycle, were identified in *Aureococcus anophagefferens* by transcriptomics analysis (Chen Q. et al., 2015). High levels of expression of genes involved in long-chain fatty acid and hydrocarbon biosynthesis were also found in *Botryococcus braunii* (Chlorophyta) under conditions of N deprivation (Fang et al., 2015). Moreover, transcription factors with driving roles in N × C interactions were shown to be associated with N stress in maize (Chen Q. et al., 2015).

It is well known that forest plantations of poplar species have large effects on C mitigation, the pulp industry, and biomass production (Studer et al., 2011). In recent years, some fast-growing tree species, such as *Populus* spp., have been widely planted worldwide (Rennenberg et al., 2010). Unlike annual plants, the yields of which are highly dependent on the addition of high-N fertilizer, perennial plants, such as trees, achieve their yields with minimal N input (20–50% less) because of their remobilization of resources, such as N in bark storage proteins (Karp and Shield, 2008). This difference exerts a large influence on life cycle studies of bioenergy chains, considering the energy consumption required to produce N fertilizer. Elucidating the genetic regulation underlying N use efficiency (NUE) (Liu et al., 2015), and identifying the important genes *via* genomic and other “-omics” approaches (Tuskan et al., 2018), will facilitate progress in genetically modified tree breeding for sustainable and efficient supply of biomass plants in the future. These investigations help maintain environmental and financial sustainability (Karp and Shield, 2008). Considering the increasingly recognized importance of forestry in ecological balance and the accelerating exhaustion of mineral resources for fertilizer, it is necessary to investigate the regulatory mechanisms and genes involved in the NUE of trees for tree improvement.

*Populus tomentosa*, also known as Chinese white poplar, is one of the fastest-growing poplar species; it is widely distributed in northern China and is of great economic and ecological importance (Du et al., 2012). *P. tomentosa* is considered a model system to explore and understand the morphological changes and molecular mechanisms of tree growth and development, as well as responses to the environment. Previously, our laboratory focused on systematically characterizing the molecular responses of *P. tomentosa* under conditions of N deficiency, including the global profiling of microRNAs (miRNAs) (Ren et al., 2015), the degradome (Chen M. et al., 2015), and long non-coding RNAs (lncRNAs) (Chen et al., 2016). It was reported that total C content, reactive oxygen species (ROS), ATP, peroxidase, superoxide dismutase (SOD), and glutamine synthetase (GS) were increased in two contrasting poplar clones *Nanlin 1388* and *Nanlin 895* (Wang X. et al., 2016). In addition, the results of transcriptomics analyses of N signaling, metabolism, and storage in poplar shoot growth and development have been reported. Transcriptomics studies showed that N starvation suppressed the expression of genes encoding most nitrate transporters (NRTs) and ammonium transporters (AMTs) in poplar leaves and genes involved in N assimilation in both roots and leaves (Luo et al., 2013). N starvation treatment was also shown to increase the fine root length and surface area, foliar starch concentration, and transcript abundance of several AMTs (AMT1;2) and NRTs (NRT1;2 and NRT2;4B) in the roots of slow-growing species (*P. popularis*) and a fast-growing species (*P. alba* × *P. glandulosa*) during acclimation to limiting N supply (Luo et al., 2013). Global transcriptomic reprogramming was shown to play a critical role underlying the physiological and morphological response of poplar leaves and roots to N starvation and excess (Luo et al., 2015). Similarly, global transcriptome reprogramming and activation of root growth were also revealed in poplar (*Populus tremula* × *Populus alba*) to low-N supply (Wei et al., 2013a). *PtaNAC1*-centered subnetwork was further revealed to be involved in increasing root biomass, which was helpful in the dynamic adjustment of poplar root architecture to low-N availability (Wei et al., 2013a,b). Further investigation demonstrated that *PtaNAC1* and *PtaRAP2.11* encoding transcription factors, F-box protein-encoding gene similar to *Hawaiian Skirt* (*PtaHWS*) had a markable influence on root development of poplar under low N (Dash et al., 2015).

Most studies to date have focused on morphological, physiological, and transcriptional changes in poplar, and few have examined the global metabolic changes in poplar combined with transcriptomics profiles under low-N conditions. To identify the metabolites and the corresponding regulatory pathways and genes involved in low-N signaling in trees, we identified genes and metabolites produced responsive to low-N stress through metabolomics and transcriptomics profile analyses. Intriguingly, we detected alterations in metabolites and transcriptional reprogramming, providing insights into the physiological and metabolic changes involved in growth and development, and obtained information to improve NUE in plantations in both agriculture and forestry.

## Materials and Methods

### Plant Materials and Treatment

*Populus tomentosa* clones (TC1521) were grown in culture on a half-strength Murashige–Skoog (MS) medium (Murashige and Skoog, 1962) (pH = 6.2) containing 20 g L^–1^ sucrose (Sigma-Aldrich, St. Louis, MO, United States) and 0.4 mg L^–1^ indole-3-butyric acid (IBA) (Sigma-Aldrich) at 25°C under a 16/8 h (day/night) photoperiod. Sixty-day-old plants were transferred into a hydroponic solution with sufficient N level for 5 days, which was changed for fresh solution every 2 days. The plants were then transferred to a solution with or without sufficient N as the control and treatment groups for 3 days as described previously (Ren et al., 2015). Briefly, plants were grown in modified half-strength mass spectrometry (MS) liquid medium (pH = 6.2) with 2 mM NH_4_NO_3_ (Sigma-Aldrich) and 1 mM KNO_3_ (Sigma-Aldrich) as sufficient N conditions (KK) (control) or with.01 mM NH_4_NO_3_ and 1 mM KCl (Sigma-Aldrich) instead of KNO_3_ for low-N treatment (DN). Whole *P. tomentosa* plants were harvested in the midmorning, immediately frozen in liquid N, and stored at −80°C.

### Metabolite Extraction

Samples were taken from six 60-day-old *P. tomentosa* plants with or without low-N treatment and ground in liquid N, and 50 ± 2.5 mg materials were transferred to 1.5-mL tubes, followed by the addition of 1 mL of 100% methanol (precooled to −20°C) and 10 mL of phenylalanine (10 μg/mL) as an internal standard, and centrifuged for 10 s. The tubes were preheated, ultrasonicated for 15 min at 60°C, and then centrifuged for 10 min. The supernatants (0.4 mL) were then transferred to 0.2 mL of acetonitrile precooled to 0°C, and 0.4 mL ultrapure water was added to the new tubes and then centrifuged for 15 min. Then, aliquots of 200 μL of the supernatants were transferred to glass bottles and dried under a gentle stream of N_2_ gas. Methoxyamine pyridine hydrochloride at a concentration of 20 mg/L (30 μL) was added to the bottles and shaken for 30 s. The oximation reaction proceeded at 37°C for 15 min. Finally, 30 μL of *N,O*-bistrifluoroacetamide (containing 1% trimethylchlorosilane) derivatization reagent was added and allowed to react for 1 h at 70°C. After these reactions, the samples were analyzed for their metabolite contents.

### Gas Chromatography-Mass Spectrometry and Metabolite Profile Analysis

Metabolites were detected by gas chromatography-mass spectrometry (GC-MS) (7890A/5975C GC-MS system; Agilent Technologies, Santa Clara, CA, United States) at Shanghai Sensichip Infotech Co. Ltd. (Shanghai, China). The Restek capillary column was an HP-5 ms (30 m × .25 mm × .25 μm) (Agilent Technologies). The parameters were as follows: injection port temperature, 280°C; EI ion source temperature, 230°C; quadrupole rod temperature, 150°C; carrier gas, high-purity helium (99.99%); splitless injection; and sample size, 1 μL. The temperature program consisted of an initial temperature of 70°C for 2 min, 10°C/min up to 320°C, and was put on hold for 6 min. GC-MS was performed by the full-scan method with a range from 50 to 550 mass-to-charge. The XC/MS software was used for metabolomics data preprocessing in the R software package (R Development Core Team, Vienna, Austria) and then compiled to remove impurity peaks due to losses from the column and the sample preparation process. The results were then organized as a two-dimensional (2D) matrix, including observation values (samples), variables (retention time/mass-to-charge ratio), and peak strength. Finally, each sample was normalized relative to the total mass using the internal standard, and the normalized data were input into SIMCA-P (ver. 11.0) for principal component analysis (PCA) using the PLS-DA model with variable importance in projection (VIP) values > 1, combined with Student’s *t*-test (*p* ≤ 0.05) to identify the differentially expressed metabolites, and searched for metabolites in the National Institute of Standards and Technology^1^ and Kyoto Encyclopedia of Genes and Genomes (KEGG)^2^ database.

### Metabolomics Data Analysis and Metabolic Pathway Construction

Before the data analysis, all data were standardized for mean-centering and unit-variance scaling using SIMCA-P with the default parameters (ver. 11.5^3^). Hierarchical clustering analysis (HCA) and PCA models were tested using all samples. Significant differences among metabolites between DN and KK were examined using the *t*-test (*p* ≤ 0.05). A heatmap was built using Pearson’s test and hierarchical clustering, performed with MATLAB 7.5 (MathWorks, Inc., Natick, MA, United States). Metabolic pathways were constructed with Metaboanalyst, and the *Arabidopsis* metabolic pathway database was used as a reference for the global algorithm. The enrichment pathways of metabolites were analyzed based on the KEGG database with a *p-*value ≤ 0.05 established as the false discovery rate (FDR) for multiple tests. The interactions among different metabolites were determined using KEGGSOAP^4^ and metabolic pathway networks were constructed using Cytoscape^5^.

### Total RNA Extraction and Illumina Sequencing Analysis

Total RNA was isolated from three 60-day-old *P. tomentosa* plants with or without low-N treatment using TRIzol reagent (Invitrogen, Carlsbad, CA, United States). The quantity and quantity of total RNA were determined using 1% agarose gel (Sigma-Aldrich) electrophoresis and an Agilent 2100 Bioanalyzer (Agilent Technologies). Complementary DNA (cDNA) libraries were constructed as described previously (Zhang et al., 2012). Briefly, total RNA was first treated with DNase I, and mRNAs were then enriched with oligo(dT) magnetic beads mixed with fragmentation buffer (Ambion, Austin, TX, United States). The fragmented messenger RNAs (mRNAs) were used to synthesize the random hexamer-primed cDNA, which was subjected to size selection and further PCR amplification. The quantity and quantity of cDNA libraries were determined with the Agilent 2100 Bioanalyzer and ABI StepOnePlus Real-Time PCR System (Applied Biosystems, Foster City, CA, United States). Finally, the cDNA libraries were sequenced using the Illumina HiSeq 2000 platform (Illumina, San Diego, CA, United States), and raw sequencing reads were processed to remove the dirty reads, i.e., reads with adapters, >5% unknown nucleotides, and low-quality reads, based on the National Institutes of Health Sequence Read Archive database (accession number: SRP063920). The obtained clean reads were applied to *de novo* assembly with Trinity^6^, which contains three independent software modules: Inchworm, Chrysalis, and Butterfly (Supplementary Figure S1). Briefly, the programs in Trinity were applied sequentially to assemble the clean reads into unique full-length transcripts, map reads into contigs, and then assemble unigenes. Finally, unigene sequences were aligned by BLASTn to the National Center for Biotechnology Information (NCBI) non-redundant nucleotide database (NT)^7^ with an *e*-value cutoff < 10^–5^. Furthermore, unigenes were aligned with the NCBI non-redundant protein database (NR) (see footnote 7) and Swiss-Prot^8^ protein database with an *e*-value cutoff < 10^–5^. To predict and classify possible functions, unigenes were also searched against the Cluster of Orthologous Groups (COG) database^9^ by BLASTx with an *e*-value cutoff < 10^–5^. Unigenes not aligned to any database were further scanned by ESTScan to obtain the amino sequences of hypothetical proteins. By blasting against these databases, four parts of the analysis of unigenes were performed: SSR analysis, unigene expression annotation, single nucleotide polymorphisms (SNP) analysis, and unigene function annotation. Further, PCA analysis and unigene expression difference analysis were conducted based on unigene expression annotation. The fragments per kilobase per million reads method was used to assess the differential expression of unigenes, as described previously (Chen et al., 2013; Hou et al., 2016), and unigenes with FDR ≤ 0.001 and |log2Ratio| ≥ 1 were regarded as differentially expressed genes (DEGs) between the DN and KK groups.

### Gene Ontology and Kyoto Encyclopedia of Genes and Genomes Analysis

Unigenes were aligned with Kyoto Encyclopedia of Genes and Genomes (KEGG)^2^ to predict the metabolic pathways, with *p* ≤ 0.05 and *q* ≤ 0.05 taken to indicate significant enrichment (Hou et al., 2016; Wang X. et al., 2016), and the functions of gene products (using BLASTx^10^) with an *e*-value cutoff < 10^–5^. Furthermore, Gene Ontology (GO) analysis was performed with NR annotation to annotate the functions of unigenes with the Blast2GO program^11^, with *p* ≤ 0.05 taken to indicate significance (Hou et al., 2016; Wang X. et al., 2016).

### Hormone Quantification by High-Performance Liquid Chromatography/Electrospray Ionization Tandem Mass Spectrometry

Samples were taken from three 60-day-old *P. tomentosa* plants with or without low-N treatment and analyzed for the concentrations of indole-3-acetic acid (IAA), abscisic acid (ABA), and gibberellic acid (GA). Extraction and purification were performed as described previously (Pan et al., 2010). Briefly, the samples were ground into a powder with a mortar and pestle, and 50-mg samples were transferred to precooled 2-mL tubes and kept in liquid N. Then, 500 μL of 2-propanol/H_2_O/concentrated HCl (2:1:0.002, vol/vol/vol) extraction solvent was added to each tube, and various volumes of internal standard solutions were added. The tubes were centrifuged at 100 rpm for 30 min on a shaker at 4°C. Then, 1 mL of dichloromethane was added to each sample and shaken for 30 min at 4°C. The tubes were further centrifuged at 13,000 × *g* for 5 min at 4°C. Then, 900 μL of solvent from the lower phase was transferred into screw-capped vials and concentrated using an N evaporator. The samples were redissolved in 100 μL of methanol. Then, 50 μL of sample solution was injected into the reverse-phase C18 Gemini high-performance liquid chromatography (HPLC) column for high-performance liquid chromatography/electrospray ionization tandem mass spectrometry (HPLC-ESI-MS/MS) analysis. Quantitative analysis of each plant hormone was performed as described previously (Pan et al., 2010).

### Real-Time Quantitative Reverse Transcription PCR to Detect the Transcripts of Differentially Expressed Genes in Response to Low-N Treatment (LN)

Samples were taken from three 60-day-old *P. tomentosa* plants with or without low-N treatment for 0, 1, 3, or 5 days as described above. Total RNA was extracted using an RNA prep Pure Plant Kit (Tiangen, Beijing, China) and reverse transcribed using a FastQuant RT Kit (With gDNase) (Tiangen). To verify RNA sequencing (RNA-Seq) profiles in this investigation, Real-Time Quantitative Reverse Transcription PCR (qRT-PCR) was performed on an Applied Biosystems 7500 Fast Real-Time PCR System using SYBR Premix Ex Taq™ (Tli RNaseH Plus; Takara, Shiga, Japan) in accordance with the manufacturer’s instructions. The primers of 14 randomly selected DEGs are listed in Supplementary Table S1. Reactions were performed in a volume of 10 μL containing 1 μL of cDNA, 5 μL of SYBR Green, 0.2 μL of forward primer, 0.2 μL of reverse primer, 0.2 μL of ROXII, and 3.4 μL of distilled water. The thermocycling conditions consisted of an initial denaturation step at 95°C for 10 min followed by 40 cycles of 95°C for 30 s, 95°C for 5 s, and 60°C for 30 s. All reactions were performed in triplicate for each gene. The 2^–ΔΔCT^ relative quantification method was used to evaluate and calculate variations (Livak and Schmittgen, 2001), with 18S rRNA used as an internal reference. The correlations of gene expression between RNA-Seq and qRT-PCR were analyzed by Pearson’s test with *p* ≤ 0.01 taken to indicate significance.

## Results

### Metabolite Profiling of *Populus tomentosa* Under N Deficiency

To determine the metabolomic regulatory mechanisms of the response of poplar to N deficiency, GC-MS was performed to analyze the changes in metabolites between *P. tomentosa* plants grown under conditions of low and sufficient N (DN and KK, respectively). The total ion chromatogram (TIC) is shown in Supplementary Figure S2. A total of 1,131 metabolites were finally identified by GC-MS (Supplementary Table S2), and partial least squares-discriminant analysis (PLS-DA) and PCA were performed to determine the accuracy and significance of differences between the KK and DN samples (Supplementary Figure S3). The KEGG enrichment analysis assigned the detected metabolites to 18 metabolic pathways, including amino acid- and sugar-related metabolism. Metabolites clustered in C fixation, starch and sucrose, and fructose and mannose metabolism were also enriched (Supplementary Table S3).

**FIGURE 1:**
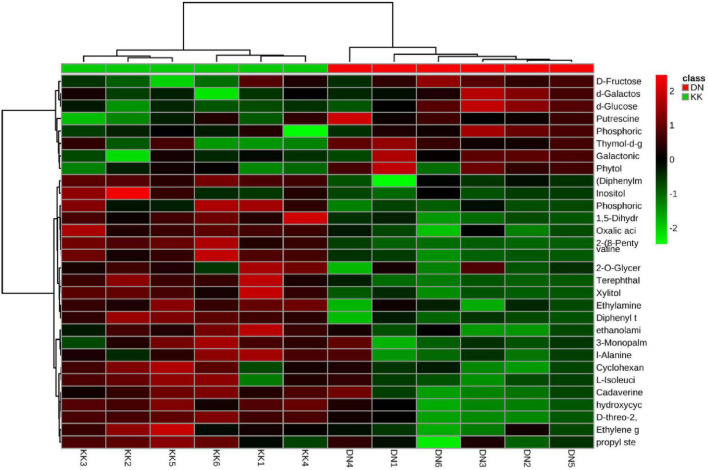
Clustering analysis of differentially expressed metabolites in *Populus tomentosa* under nitrogen (N) deficiency. KK1, KK2, KK3, KK4, KK5, and KK6 represent six replicates of control samples, while DN1, DN2, DN3, DN4, DN5, and DN6 represent six replicates of low-N treatment samples.

Among the 1,131 metabolites, 30 with significantly differential expression were identified by PLS-DA with VIP > 1 and *p* ≤ 0.05 (Figure 1). Most of these metabolites (70%) were significantly suppressed under conditions of low-N stress. Marked reductions in the levels of metabolites with amino groups (such as valine, L-isoleucine, L-alanine, cadaverine, ethylamine, and ethanolamine) were assumed to result in the cessation of the de novo synthesis of the free amino acids because of N deficiency (Table 1). Furthermore, the levels of alcohols were reduced under low-nitrate stress, as shown by the *a*-hydroxycyclohexene, cyclohexanol, ethylene glycol, inositol, and xylitol contents compared with the controls. However, N deficiency led to the accumulation of some soluble sugars, including D-fructose, D-galactose, and D-glucose, in *P. tomentosa* plants.

**TABLE 1:** Identification of changed metabolites in *Populus tomentosa* under N deficiency.

Metabolites	DN	KK	*p*-value	Fold change
Xylitol	91.93	488.77	0.01	–2.52
(Diphenylmethylene)(1-mesitylethyl) azane oxide	6.36	24.24	0.00	–1.93
Valine	80.97	202.64	0.00	–1.32
Terephthalic acid	19.14	43.26	0.00	–1.18
Diphenyl terephthalate	32.26	72.20	0.00	–1.16
2-(8-Pentyldodecahydropyrrolo[1,2-a] quinolin-3-yl) ethanol	61.45	129.38	0.00	–1.07
L-isoleucine	62.09	105.46	0.02	–0.76
L-alanine	594.97	962.70	0.02	–0.69
3-Monopalmitin ether	29.16	45.01	0.04	–0.63
1,5-Dihydroxy-6-methoxyxanthone	326.85	492.94	0.00	–0.59
Cadaverine	1343.61	2024.62	0.01	–0.59
Ethylamine	23.51	34.14	0.00	–0.54
Propyl stearate	81.68	114.46	0.05	–0.49
Cyclohexanol	45.99	64.14	0.01	–0.48
D-threo-2,5-hexodiulose	228.08	314.37	0.00	–0.46
A-hydroxycyclohexene	279.35	367.96	0.00	–0.40
Inositol	44.08	56.51	0.04	–0.36
Ethylene glycol	245.87	311.93	0.02	–0.34
Phosphoric acid	3664.68	4563.56	0.01	–0.32
2-*O*-glycerol-*a*-D-galactopyranoside	226.28	277.92	0.05	–0.30
Ethanolamine	232.47	278.27	0.01	–0.26
D-galactose	79.10	70.28	0.04	0.17
D-glucose	1299.78	1108.73	0.01	0.23
D-fructose	590.29	437.26	0.04	0.43
Putrescine	22.08	16.19	0.05	0.45
Phytol	137.39	96.67	0.03	0.51
Phosphoric acid propyl ester	48.54	27.87	0.04	0.80
Galactonic acid	24.84	14.24	0.02	0.80
Thymol-*a*-D-glucopyranoside	67.09	36.97	0.02	0.86
Oxalic acid	139.16	306.90	0.00	1.14

### Network Construction of Responsive Metabolites in *Populus tomentosa* Under N Deficiency

**FIGURE 2:**
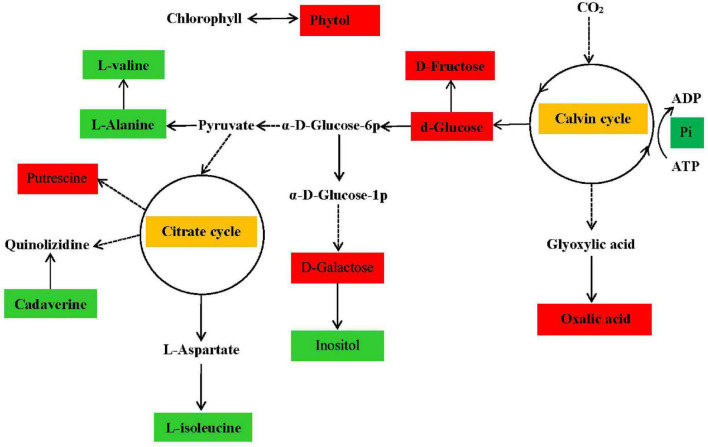
Metabolic pathways showing the most primary metabolites in *P. tomentosa* under N deficiency. Major metabolites were enriched in the citrate and Calvin cycles. Solid arrows indicate direct transformation and dashed arrows indicate indirect or complicated connections. Metabolites indicated by red and green rectangles represent increased and decreased concentrations in the low-N treatment group, respectively.

Based on the pathways of low-N-responsive metabolites, we further constructed metabolic networks to analyze their interactions (Figure 2). As shown in the metabolic networks, the induced sugars, including D-glucose, produced by the Calvin cycle influenced the synthesis of diverse downstream metabolites, including amino acids and putrescine belonging to the citrate cycle. Interestingly, in addition to the induced D-glucose, D-fructose, and D-galactose, other sugar-related metabolites, such as galactonic acid and oxalic acid, were enhanced under conditions of N deficiency in this study. The induced level of sugar may have been due to reduced carbohydrate metabolism during degradation and utilization by downstream metabolites of amino acids derived from N assimilation, in turn, due to low-N stress, consistent with the pattern of N assimilation-related gene regulation revealed by transcriptomics analysis, which also revealed the connection of C and N metabolism.

### RNA-Seq of *Populus tomentosa* Under N Deficiency and Gene Function Annotations

To further determine the genes involved in regulating low-N signaling in poplar under conditions of N deficiency, RNA-Seq was conducted in DN and KK *P. tomentosa* plants. After discarding the contaminated raw data, 104,843,476 clean reads (52,903,032 reads from DN, 51,940,444 reads from KK) containing 943,591,2840 nt were obtained (Supplementary Table S4). Based on these clean reads, 154,857 and 146,823 contigs of the treatment and control, respectively, were further assembled to 71,801 (DN) and 82,908 (KK) unigenes with mean lengths of 582 and 508 nt, respectively (Supplementary Table S5).

The unigene sequences were then aligned to the NCBI NR protein database, Swiss-Prot, KEGG, and COG by BLASTx with an *e*-value cutoff < 10^–5^, and to the NCBI NT nucleotide database by BLASTn with an *e*-value cutoff < 10^–5^, to retrieve proteins with the highest sequence similarity to the given unigenes along with their protein functional annotations (Supplementary Figure S1). For function annotation analysis, we obtained 52,816, 54,584, 32,342, 29,222, 18,431, and 42,264 unigenes, which were annotated to the NR, NT, Swiss-Prot, KEGG, COG, and GO databases, respectively, and a final total of 59,125 unigenes were annotated (Supplementary Tables S6, S7).

**FIGURE 3:**
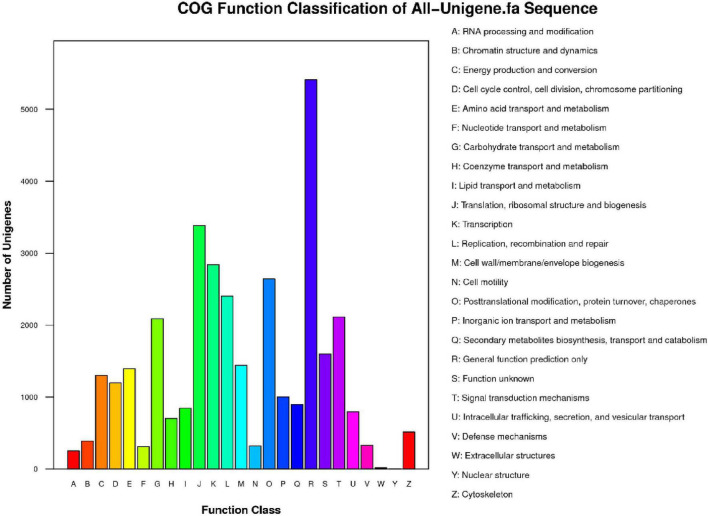
Cluster of Orthologous Groups (COG) function categories of unigenes in *P. tomentosa*. COG function classes are shown on the *x*-axis and the full names of the functions are annotated on the right. The numbers of unigenes in one class are shown on the *y*-axis.

Then, we searched the unigene sequences against the COG database to predict the possible functions and understand the global gene function distributions of the species. A total of 18,431 sequences from 59,125 unigenes were mapped to the COG database; among the 25 categories, the largest group of unigenes (*n* = 5,411, 29.36%) were annotated as “General function prediction only,” followed by “Translation, ribosomal structure and biogenesis” (*n* = 3,384, 18.36%) and “Transcription” (*n* = 2,840, 15.41%), while genes functioning in “Nuclear structure” (*n* = 2, 0.01%) and “Extracellular structures” (*n* = 17, 0.09%) represented the smallest categories (Figure 3).

### Global Transcriptomic Changes of *Populus tomentosa* Under N Deficiency

**FIGURE 4:**
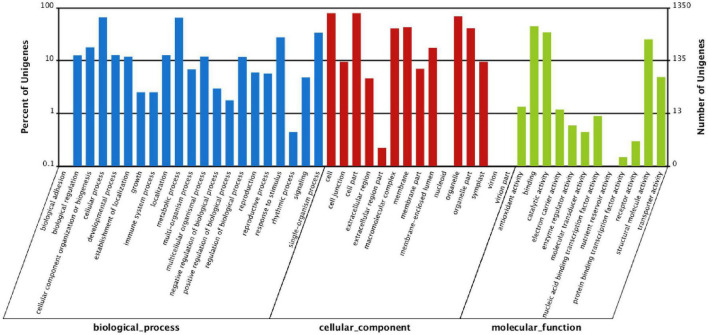
The Gene Ontology (GO) classification of differentially expressed genes (DEGs; DN/KK) in *P. tomentosa* under conditions of N deficiency. The percentage of unigenes in one class is shown on the *y*-axis, while DEGs under DN treatment were classified into three classes (biological processes, cellular component, molecular function) on the *x*-axis.

To identify the genes involved in the response of *P. tomentosa* to low-N stress, we further screened unigenes with significant changes based on the assembled data. A total of 1,561 downregulated and 1,101 upregulated DEGs were identified with a false discovery rate (FDR) ≤ 0.001 and | log2Ratio| ≥ 1 (Supplementary Table S8a and Supplementary Figure S4). To exclude statistical error, more rigorous criteria (fragments per kilobase per million reads of unigene in KK and DN > 1) were applied, and 1,017 DEGs were finally identified under conditions of N deficiency in *P. tomentosa* (Supplementary Table S8b). To understand the functions of these genes in the N stress response, we further aligned DEGs to the GO database. The results revealed 21, 15, and 12 classes of DEGs involved in biological process, cellular component, and molecular function, respectively (Figure 4). In the biological process, most DEGs fell into the cluster of “cellular process” (889, 1.50%), “metabolic process” (874, 1.48%), “single-organism process” (454,.77%), and “response to stimulus” (373,.63%). Three categories of DEGs, “organelle” (*n* = 931, 1.57%), “cell” (*n* = 1,072, 1.81%), and “cell part” (*n* = 1,072, 1.81%), were dominant in the cellular component, whereas “binding” (*n* = 604, 1.02%), “catalytic activity” (*n* = 464, 0.78%), and “structural molecule activity” (*n* = 342, 0.58%) represented the main DEG groups in molecular function (Supplementary Table S9).

To further understand the molecular pathways in which these *P. tomentosa* genes were involved, we scanned the DEGs into the KEGG database. As a result, 1,329 genes were finally annotated to 104 KEGG pathways, among which 16 pathways were significantly enriched at *p* ≤ 0.05, including “genetic information processing,” “cellular processes,” “metabolism,” “environmental information processing,” and “organismal systems” under conditions of low-N stress in *P. tomentosa* (Table 2). Intriguingly, five pathways related to amino acid metabolism were significantly enriched and 20 DEGs were involved in N metabolism, indicating that *P. tomentosa* shows a marked response to low-N stress. On the other hand, four pathways related to carbohydrate metabolism were also enriched, i.e., amino sugar and nucleotide sugar metabolism, inositol phosphate metabolism, pentose and glucuronate interconversion, and propanoate metabolism, suggesting that carbohydrate regulation is involved in the response to low-N stress (Table 2). In addition, many DEGs could be clustered into several metabolic pathways, including N metabolism, auxin-related pathway, or function as some transporters and kinases, etc. (Supplementary Table S10).

**TABLE 2:** Kyoto Encyclopedia of Genes and Genomes (KEGG) pathway annotation of *Populus tomentosa*.

Pathway	DEGs (1329)	All genes (29,222)
Ribosome	471 (35.44%)	1661 (5.68%)
Phagosome	44 (3.31%)	450 (1.54%)
Endocytosis	64 (4.82%)	896 (3.07%)
Nitrogen metabolism	20 (1.5%)	190 (0.65%)
Arginine and proline metabolism	21 (1.58%)	252 (0.86%)
Biosynthesis of unsaturated fatty acids	11 (0.83%)	103 (0.35%)
Beta-alanine metabolism	13 (0.98%)	141 (0.48%)
Histidine metabolism	9 (0.68%)	87 (0.3%)
Amino sugar and nucleotide sugar metabolism	25 (1.88%)	354 (1.21%)
Inositol phosphate metabolism	17 (1.28%)	219 (0.75%)
Tryptophan metabolism	12 (0.9%)	139 (0.48%)
Pentose and glucuronate interconversions	23 (1.73%)	345 (1.18%)
Steroid biosynthesis	9 (0.68%)	104 (0.36%)
Lysine degradation	10 (0.75%)	121 (0.41%)
Propanoate metabolism	15 (1.13%)	207 (0.71%)
Phosphatidylinositol signaling system	15 (1.13%)	207 (0.71%)

### Genes Related to N Acquisition, Allocation, and Assimilation in Response to Low-N Stress

Plants take up N from two sources in the soil, ammonium, and nitrate. Nitrate is reduced to nitrite (NO_2_^–^) and then ammonium, which is incorporated into glutamate and glutamine with a net supply of 2-oxoglutarate (provided by isocitrate dehydrogenase, IDH), catalyzed by the GS/glutamate synthase (GOGAT) cycle. Other amino acids, such as aspartate and asparagine, are then generated by aspartate aminotransferase (*AspAT*) and asparagine synthetase (*AS*) (Suárez et al., 2002). In the nitrate assimilation process, N is incorporated into N-containing compounds, such as other amino acids, chlorophylls, and nucleic acids. Analysis of the transcriptome data revealed downregulation of N acquisition genes, such as putative nitrate reductase (NR; twofold downregulated) and GS (1.60-fold downregulated). The genes that participate in the biogenesis of various amino acids and other N-containing compounds from glutamine/glutamate were shown to be downregulated: putative IDH (11.60-fold downregulated), AspAT (1.17-fold downregulated), and AS (1.37-fold downregulated) (Supplementary Table S10 and Table 3).

**TABLE 3:** Differentially expressed genes (DEGs) identified by GO and KEGG involved in N metabolism in *Populus tomentosa*.

Gene ID	log_2_Ratio (DN/KK)	Regulation (DN/KK)	Annotation
CL8615.Contig2_All	–1.61	Down	Aminomethyltransferase, mitochondrial
Unigene23815_All	–1.34	Down	Asparagine synthase
CL6264.Contig2_All	–1.07	Down	Carbonic anhydrase, chloroplastic
Unigene30284_All	2.15	Up	Cytochrome b5
Unigene29769_All	2.77	Up	Cytochrome b-c1 complex subunit Rieske-3, mitochondrial
Unigene35581_All	–11.65	Down	Cytochrome c1–1, heme protein, mitochondrial
Unigene31830_All	12.10	Up	Cytochrome c1–2, heme protein, mitochondrial
Unigene24651_All	–1.05	Down	Ferredoxin–nitrite reductase, chloroplastic
CL4868.Contig1_All	1.01	Up	Glutamate dehydrogenase 1
Unigene30445_All	3.65	Up	Glutamate dehydrogenase 3
Unigene31623_All	3.92	Up	Glutamate dehydrogenase 3
Unigene22953_All	–1.60	Down	Glutamine synthetase, chloroplastic
Unigene27220_All	–1.37	Down	Glutamine synthetase, chloroplastic
Unigene38713_All	–12.18	Down	NADP-specific glutamate dehydrogenase
Unigene18435_All	–3.30	Down	Nitrate reductase
CL4201.Contig1_All	3.05	Up	Nitrate reductase (NADH)
CL933.Contig1_All	–2.55	Down	Nitrate reductase (NADH)
CL933.Contig2_All	–2.14	Down	Nitrate reductase (NADH)
CL933.Contig3_All	–2.22	Down	Nitrate reductase (NADH)
Unigene34301_All	–11.88	Down	Nitrate reductase (NADH)
Unigene38634_All	–12.26	Down	Nitrate reductase (NADH)

*DN, low N treated; KK, control. NADP, nicotinamide adenine dinucleotide phosphate; NADH, nicotinamide adenine dinucleotide.*

Consistent with these observations, several genes encoding putative amino acid synthesis-related proteins were suppressed under low-N conditions, such as proline-rich protein (*CL2231.Contig3_Al*l), aspartate aminotransferase (*CL8615.Contig2_All*), cationic amino acid transporter (*Unigene38715_All*), and GS (*Unigene27220_All*, *Unigene22953_All*) (Supplementary Table S10). These results indicated the decreased level of N assimilation and further utilization under conditions of N deficiency stress in *P. tomentosa*.

### Interaction of C and N Metabolism Under Conditions of N Deficiency

C assimilation and N absorption control the status of plant growth and development. Extensive studies have shown the interdependence and interaction of C and N metabolism in photosynthesis, amino acid metabolism, lipid metabolism, and carbohydrate metabolism, including the TCA cycle, Calvin cycle, glycolysis, etc. (Sinha et al., 2015; Goel et al., 2016). Briefly, C metabolism is mostly dependent on photosynthesis, in which N participates in the biosynthesis of chlorophyll and other related proteins. Correspondingly, N assimilation demands energy and C skeletons from C metabolism.

Obviously, a low-N environment affects the functions of the chloroplasts and chlorophyll, and putative related proteins were downregulated in *P. tomentosa*, such as chlorophyll a–b binding protein of light-harvesting complex (LHC) II type I (2.73-fold downregulated), chlorophyll a–b binding protein CP26 (1.21-fold downregulated), photosystem I reaction center subunit psaK (1.37-fold downregulated), oxygen-evolving enhancer protein 2 of photosystem II (1.89-fold downregulated), ferredoxin–NR (1.05-fold downregulated), and chloroplast processing peptidase, indicating depressed photosynthesis capacity under low-N conditions (Supplementary Table S10).

On the other hand, our transcriptomics data revealed significant changes in the enzymes involved in the TCA cycle, glycolysis, and lipid degradation. Particularly, three enzymes involved in glycolysis, i.e., 6-phosphofructokinase 4, fructose-bisphosphate aldolase, and phosphoglycerate mutase, were decreased under low-N conditions. Moreover, five enzymes in the TCA cycle were altered in *P. tomentosa* under conditions of low-N stress, and genes encoding putative citrate synthase, succinyl-CoA synthetase, and succinate dehydrogenase were upregulated, while genes encoding putative aconitate hydratase, IDH (time-limiting enzyme), and PEPCase (flux regulator of TCA) were downregulated. Conversely, genes encoding five putative enzymes involved in lipid degradation were induced, i.e., stearoyl-CoA desaturase, 3-hydroxyacyl-CoA dehydrogenase, acetyl-CoA acyl-transferase, fatty acyl-CoA reductase, and long-chain-aldehyde dehydrogenase (Supplementary Table S10). Our data indicated decreased energy from glucose and increased energy and C skeletons from lipid, while the downregulation of putative phosphoenolpyruvate carboxylase (PEPCase) and IDH depressed the flux of oxaloacetate into the TCA cycle and lowered the production of ATP and nicotinamide adenine dinucleotide (NADH) in plants, respectively. Taken together, these results suggest reduced carbohydrate metabolism and energy production, particularly decreased energy generated from glucose in *P. tomentosa*, under conditions of low-N stress.

### Hormone Signaling-Related Gene Profiles and Hormone Quantification

**FIGURE 5:**
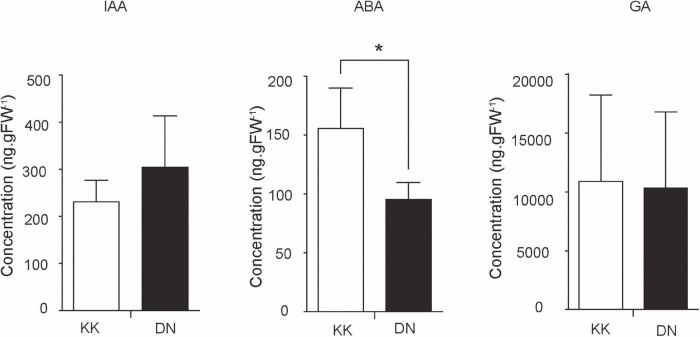
Quantification of the concentrations of gibberellic acid (GA), abscisic acid (ABA), and indole-3-acetic acid (IAA) in *Populus tomentosa* plants between control (KK) and low-N treatment (DN) groups. The *y*-axis represents the hormone concentration, while the *x*-axis represents the KK and DN samples. *denotes significant difference (*p* ≤ 0.05).

Auxin and ABA were reported to be closely related to N signaling (Sakakibara, 2003; Mockaitis and Estelle, 2008; Lu et al., 2015). The plant hormones IAA, ABA, and GA in *P. tomentosa* were quantified by HPLC-ESI-MS/MS in this study. The concentration of IAA was increased under low-N conditions (1.52) compared with normal conditions (1.16) (Figure 5). The accumulation of IAA was consistent with the increased expression of genes encoding auxin response genes. For example, putative *ARF6* (1.50-fold increase) and *IAA10* (2.23-fold increase) were markedly induced (Table 4).

**TABLE 4:** Hormone-related DEGs in *Populus tomentosa* under N deficiency.

Gene ID	Log_2_Ratio (DN/KK)	Regulation (DN/KK)	Annotation
Unigene21573_All	1.50	Up	Auxin response factor 6
CL5591.Contig2_All	2.23	Up	Auxin-responsive protein IAA10
Unigene40069_All	–11.57	Down	IAA-amino acid hydrolase ILR1-like 1
Unigene9971_All	–1.43	Down	IAA-amino acid hydrolase 11 (ILL11)
Unigene36745_All	–1.77	Down	UDP-glycosyltransferase 76C3
Unigene28931_All	12.09	Up	Isopentenyl diphosphate isomerase
Unigene39732_All	–11.54	Down	Similar to geranylgeranyl hydrogenase
Unigene5624_All	1.12	Up	Geranylgeranyl reductase
Unigene40194_All	–12.20	Down	RAB proteins geranylgeranyl transferase component A
Unigene39275_All	–11.71	Down	RAB proteins geranylgeranyl transferase component A

*RAB proteins, Ras -Associated binding protein.*

On the other hand, the concentrations of ABA and GA were generally reduced, especially ABA, with more significant repression under conditions of low-N stress (Figure 5). ABA and GA are biosynthesized in the methylerythritol-4-phosphate (MEP) pathway. In the network, the conversion between isopentenyl pyrophosphate (IPP) and dimethylallyl diphosphate (DMAPP) is catalyzed by putative isopentenyl diphosphate isomerase (*IDI*; 12.09-fold). Next, isoprenyl pyrophosphate synthase (*IPPS*) catalyzes IPP to generate isopentenyl AMP (iAMP), the precursor of cytokinin (CTK). Meanwhile, IPP and DMAPP can be transformed to geranylgeranyl diphosphate (GGDP), further generating GA and other carotenoids. In addition, GGDP can be reduced to phytyl diphosphate by putative geranyl-geranyl reductase (1.12-fold), thus providing phytol (0.507) for chlorophyll synthesis (Table 4). The concentrations of GA and ABA in this pathway showed small reductions, whereas another route of phytol utilization was increased under conditions of N deficiency.

### Detection of Dynamic Expression of Differentially Expressed Genes by Real-Time Quantitative Reverse Transcription PCR

**FIGURE 6:**
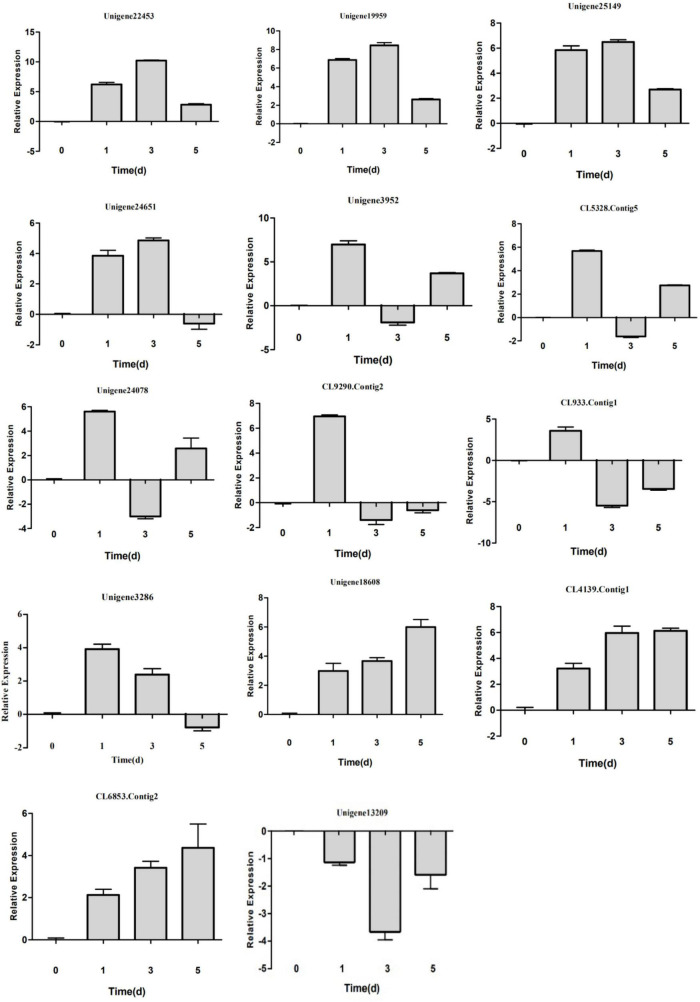
Detection of DEG expression in *Populus tomentosa* under control (KK) and low-N treatment (DN) conditions by real-time quantitative reverse transcription-polymerase chain reaction (qRT-PCR). The *x*-axis represents the treatment time (0, 1, 3, and 5 days), while the *y*-axis represents relative gene expression level.

To validate the expression profiles of the identified DEGs and determine their possible dynamic responses to low-N stress at different treatment stages, the levels of expression of 14 DEGs were investigated by qRT-PCR after 0, 1, 3, and 5 days of low-N treatment (Figure 6). Except for *Unigene24651*, *Unigene3952*, *CL5328.Contig5*, and *CL4139.Contig1*, the expression changes of most (10) DEGs at 3 days under low-N treatment conditions quantified by qRT-PCR were consistent with the abundance determined by RNA-Seq analysis. The inconsistencies in the expression of the four genes quantified by these two methods may have been due to insufficient coverage sequencing depth to reflect the true distribution of these genes or to differences in the data normalization criteria for the two methods. Further expression correlation analyses of these ten DEGs demonstrated that the expression determined by RNA-Seq was positively correlated with that revealed by qRT-PCR (Supplementary Figure S5).

After further examining the expression changes by qRT-PCR, we found three different expression patterns (Figure 6). First, the expression abundance increased gradually after 1 and 3 days of low-N treatment, peaked at 3 days, and then declined steadily at 5 days for *Unigene 22453*, *Unigene19959*, *Unigene25149*, and *Unigene24651*. It is worth noting that *Unigene24651* was markedly repressed at 5 days relative to 0 days, in contrast to *Unigene 22453*, *Unigene19959*, and *Unigene25149*. Second, the expression levels of *Unigene3952*, *CL5328.Contig5*, *Unigene24078*, *Unigene3286*, *CL933.Contig 1*, and *CL9290.Contig2* peaked after 1 day of low-N treatment and decreased after 3 days. The expression levels of *Unigene3952*, *CL5328.Contig5*, and *Unigene24078* were increased, while those of *CL933.Contig 1* and *CL9290.Contig2* were reduced, after 5 days. Both of these patterns showed the dynamic fluctuation of miRNA expression in *P. tomentosa* in response to low-N stress at different stages. Third, *Unigene18608*, *CL6853.Contig2*, and *CL4139.Contig1* were all induced, while *Unigene13209* was repressed, at all stages of low-N stress.

### Correlation of Gene Expression and Metabolic Changes Under Low-N Stress

Based on the global metabolome, we found an array of primary metabolites with differential changes under DN treatment, meanwhile, the transcriptome data showed accompanied changes of genes, which were involved in biosynthesis or degradation of these key metabolites. Thereafter we compared the expression of inducement or suppression of genes with these corresponding metabolites. Coordinated metabolomics and transcriptomics analyses revealed the relative expression of five pairs of metabolites and unigenes (Table 5). Our results revealed five genes encoding enzymes involved in the biogenesis or degradation of associated carbohydrates, and genes and metabolites showed a co-expression pattern. The data indicated that the accumulation of D-glucose and galactonic acid could be attributed to their increased biosynthesis, catalyzed by dTDP-D-glucose 4,6-dehydratase and β-galactosidase, respectively, while the accumulation of D-fructose and D-galactose may have been the result of a reduction of fructose-2,6-bisphosphatase and galactose mutarotase expression.

**TABLE 5:** Coordinated changes in gene expression that led to alternation of some key primary metabolites in *Populus tomentosa* under N deficiency.

Metabolite	Log_2_ (DN/KK)	Gene annotation	Gene ID	Function	GO molecular function
D-glucose	0.23	dTDP-D-glucose 4,6-dehydratase	Unigene29785_All (4.38),	Biosynthesis	Oxidoreductase activity
			Unigene33572_All (2.54)		
Galactonic acid	0.80	Beta-galactosidase	CL1096.Contig2_All (0.95),	Biosynthesis	Beta-galactosidase activity
			CL1096.Contig3_All (0.95),		
			CL1096.Contig4_All (0.95), CL1096.Contig5_All (1.19),		
			CL1096.Contig6_All (0.95), CL1096.Contig7_All (0.12),		
			CL1096.Contig8_All (0.69), CL1096.Contig9_All (0.69),		
D-fructose	0.43	Fructose-2,6-bisphosphatase	CL4139.Contig1_All (−1.15),	Degradation	Phosphoglycerate mutase activity
			CL5072.Contig1_All (−0.17),		
			CL5072.Contig2_All (0.03), CL5072.Contig3_All (−0.11),		
Inositol	−0.36	Inositol oxygenase 2	Unigene29428_All (1.07),	Degradation	Inositol oxygenase activity
			CL1004.Contig2_All (0.01),		
D-galactose	0.17	Galactose mutarotase and related enzymes	CL4080.Contig1_All (−1.34),	Degradation	Aldose 1-epimerase activity
			CL1004.Contig3_All (−0.63),		
			CL1004.Contig4_All (−1.19)		
			Unigene34048_All (0.10),		
			Unigene34101_All (−0.48), Unigene38082_All (−0.55)		

## Discussion

Due to their ability to perform high-throughput genome-wide characterization, transcriptomics analyses have been extensively applied to systemically investigate the genes responsible for the development and adaptation of plants to different environmental stresses (Vogel et al., 2016; Wang Y. N. et al., 2016; Woo et al., 2016). It is well known that N, one of the most important nutrients, functions as a component of various key cell molecules, such as proteins (amino acids), nucleic acids, chlorophyll, and secondary metabolites. Consequently, many studies have focused on the mechanisms underlying the roles of N in plants, and DEGs in response to N deficiency. Several genes involved in N assimilation and utilization have been identified in a variety of plants, including cucumber (Zhao et al., 2015), rice (Yang et al., 2015), maize (Chen Q. et al., 2015), *Medicago truncatula* (Bonneau et al., 2013), and *Arabidopsis* (Günther et al., 2012).

### Global Changes in the Transcriptomic Network of *Populus tomentosa* Under N Deficiency

The results of the present study showed that N deficiency leads to the differential expression of many genes, depresses N assimilation, reduces amino acid biosynthesis, and reduces photosynthetic and energy production capacity. Similar to previous studies, a large number of DEGs were identified in *P. tomentosa* in response to low-N conditions, including several genes directly related to N metabolism, encoding putative nitrate reductase (*NR*), glutamate dehydrogenase (*GDH*), glutamine synthetase (*GS*), among others (Supplementary Table S10). Generally, plants can directly use ammonium, but not nitrate (Jackson et al., 2008). Nitrate first must be reduced by NR (Bowsher et al., 1988), which is then reduced to ammonium by the concerted action of NR (Foyer and Mullineaux, 1998). With the action of two enzymes, GOGAT and GS, ammonium can form the amino acids that combine with the organic acids produced by photosynthesis.

This study showed that five transcripts of putative NR and two genes encoding putative GS were downregulated under conditions of low-N stress because of N source limitations (Supplementary Table S10). However, the expression levels of *NR* and *GS* in *Arabidopsis* and rice were elevated under low-N conditions, possibly indicating differences in the mechanisms regulating N uptake and accumulation among *P. tomentosa*, *Arabidopsis*, and rice. A previous investigation revealed direct coupling of N assimilation and photosynthesis in chloroplasts, in a mechanism designated as nitrate photo assimilation (Bot et al., 2009). Several studies have revealed universal physiological and metabolic changes involved in the responses of plants to N deficiency, including significant reductions of chlorophyll content, growth, and photosynthesis, protein, starch, etc. (Mesnard and Ratcliffe, 2005). We detected four decreased transcripts of putative chlorophyll a–b binding proteins, indicating downregulated photosynthesis under low-N conditions. Similarly, previous investigations showed that genes involved in photosynthesis were downregulated in the leaves of *Arabidopsis* (Liu et al., 2017) and roots of rice (Cai et al., 2012).

### Responses of Hormone and Hormone-Related Genes to N Deficiency

Among plant hormones, auxin and ABA have been reported to be closely related to N signaling (Sakakibara, 2003; Lu et al., 2015; Yu et al., 2016). Our results demonstrated the interaction of GA signaling with N deprivation. Transcripts of putative GA-insensitive (*GAI*, a DELLA domain-containing GRAS family transcription factor) were induced (1.57-fold upregulation) and putative repressor of GA1-3-like 1 (*RGL1*) was suppressed (1.78-fold downregulation) under conditions of low-N stress. In cucumbers, suboptimal root zone temperatures were shown to suppress GA biogenesis and plant growth, while exogenous GA restored seedling biomass and enhanced N uptake (Bai et al., 2016). These results show the interdependent promotional role between GA biogenesis and N acquisition.

In *Arabidopsis*, N deprivation induced an increase in auxin content and expression of the auxin synthetic gene, *TAR2*, and the expression of auxin influx carriers (AUX/LAX family, such as *AUX1*, *LAX1*, *LAX2*, *LAX3*) and efflux transporter factors (PIN and PGP, e.g., *PIN1*, *PIN2*, and *PIN4*) could be regulated by the nitrogen/carbon (N/C) ratio (Gutiérrez et al., 2007). In addition, auxin signaling is related to N availability, and *ARF8* could be induced by nitrate deficiency. miR160 has been shown to suppress *ARF10*, *ARF16*, and *ARF17*, while miR167 targets *ARF6* and *ARF8* (Rhoades et al., 2002). In maize, low N supply increased the levels of root auxin and NO, and then enhanced root elongation (Mi et al., 2008). Auxin was shown to trigger the accumulation of miR393, leading to the repression of *TIR1*/*AFBs*, depressed auxin perception and response, and ultimately, homeostasis (Chen et al., 2011). In addition, by binding to auxin and AUX/IAA, TIR1 could direct the ubiquitination and degradation of AUX/IAA proteins. On the other hand, binding to AUX/IAA promotes the activation of ARFs and the depression of other early genes in the auxin response pathway (Mockaitis and Estelle, 2008). Studies of *Arabidopsis* have revealed that the coordination of miRNA nodes directs the nitrate and auxin signaling involved in lateral root formation. Both miR393 and its target, *AFB3*, are known to be induced by nitrate and glutamine/glutamate, which affect the uptake of auxin (Vidal et al., 2010). However, miR167 is depressed under conditions of nitrate treatment, thus causing the accumulation of *ARF8* and the downstream gene product, *GH3*, leading to a homeostatic level of auxin and modulation of the root architecture (Yang et al., 2006). Previous studies have validated the interaction of pto-miR160a and its target mRNA, *pto-ARF16*, which are involved in tree growth (Tian et al., 2016). Our previous investigation demonstrated the downregulation of pto-miR160f, pto-miR167a/b, pto-miR167c–f, pto-miR167g, and pto-miR390a–d and the upregulation of pto-miR393a/b under conditions of low-N stress in *P. tomentosa*, and thus the association of nitrate with auxin signaling (Ren et al., 2015). Furthermore, our transcriptomics data showed the corresponding induction of putative *pto-IAA10* (*CL5591.Contig2_All*, 2.2-fold upregulation) and *pto-ARF6* (*Unigene21573_All*, 1.5-fold upregulation), indicating that the nitrate-induced auxin signaling pathway plays a role in root architecture plasticity.

Previous studies provided evidence that the biosynthesis of CTK is dependent on N status to induce IPT genes, and that nitrate and glutamine act as inducers in *Arabidopsis* (Takei et al., 2004) and rice (Kamada-Nobusada et al., 2013). Similar to ABA, reduced CTK in a low-N environment was reported to enhance lateral root formation (Kiba et al., 2010). In addition, cytokinin-*N*-glucosyltransferase was shown to be involved in CTK homeostasis in *Arabidopsis*. Our study detected the downregulation of putative cytokinin-*N*-glucosyltransferase (1.77-fold downregulation), consistent with the results seen in *Arabidopsis* under conditions of N starvation (Bi et al., 2007) and rice (Cai et al., 2012). By catalyzing *N*-glucosylation of CTKs, CTKs then regulated CTK-response genes, such as *CKX3*, *AHK2*, *AHK3*, *ARR1*, and *LOG2* (Wang et al., 2013), thus controlling the response to N deficiency in *P. tomentosa*. In addition, ethylene response regulator was increased in *Botrytis cinerea*-infected *Solanum lycopersicum* under nitrate-limiting conditions, suggesting that N status may be related to pathogen susceptibility (Vega et al., 2015). As a consequence, diverse plant hormones should be involved in the adaptation of *P. tomentosa* to conditions of low-N stress.

### Global Changes of Metabolic Networks in Response to N Deficiency

To investigate the effects of low-N stress on the metabolic regulation of *P. tomentosa* and validate gene expression at the level of the transcriptome, GC-MS was further applied for identification and robust quantification of primary metabolites in plant samples, including sugars, sugar alcohols, amino acids, organic acids, and polyamines. These results should enable a comprehensive evaluation of metabolic changes in *P. tomentosa* exposed to low-N stress, and highlight fields of interest for future studies of the NUE of poplar. The contents of amino acids were changed markedly under conditions of low N supply, including valine (1.32-fold downregulation), L-alanine (0.69-fold downregulation), and L-isoleucine (0.76-fold downregulation), similar to the gene expression pattern of the transcriptome. These changes were somewhat different from those seen in previous studies related to N starvation. In tomatoes, the L-alanine level was reported to decrease to less than half that recorded at the start of the experiment, while L-isoleucine and valine did not change markedly (Urbanczyk-Wochniak and Fernie, 2005). Tschoep et al. (2009) found that a low-N growth regime caused shoot growth reduction and increased the levels of many amino acids. The levels of amino acids, including alanine, serine, glycine, valine, leucine, isoleucine, phenylalanine, and tyrosine, derived from pyruvate and phosphoenolpyruvate increased in *Synechocystis* under conditions of N deficiency (Tschoep et al., 2009). This may have been related to differences between the species or experimental conditions. Urbanczyk-Wochniak and Fernie (2005) reported that different environmental factors could cause different changes in amino acids, although the variation of amino acids was not linear under low-N conditions, with some amino acids showing a marginal decline initially but an increased level at the end of the experiment. The results of this investigation were also somewhat consistent with previous studies of the poplar transcriptome, which indicated that poplar species could slow down N assimilation under conditions of limited N supply (Luo et al., 2013).

In this study, the downregulation of genes participating in the glyoxylate cycle [isocitrate lyase, malate synthase, and phosphoenolpyruvate carboxykinase (ATP: oxaloacetate carboxy-lyase (transphosphorylation), EC 4.1.1.49] may have had adverse effects on the growth of *P. tomentosa*, indicating a depressed state of C metabolism providing less carbon (by synthesizing a variety of carbohydrates) and energy (providing NADPH and then ATP) for plant growth and development. On the other hand, N and C metabolism could be mutually affected due to the requirement for C skeletons for the production of N metabolites, such as amino acids. In dicots, nitrate levels could affect carbohydrate metabolism (Scheible et al., 1997). Phloem transport and C export are suppressed under low-N conditions (Nunes-Nesi et al., 2010). The significantly altered amino acid production seen in this study was related to pyruvate recycling. The accumulation of foliar starch was also found in *Arabidopsis* and maize exposed to low-N conditions due to the reduced demand of C skeletons for N compounds, including amino acids and proteins (Ikram et al., 2012; Schlüter et al., 2012). Concerning C metabolism, the expression of some sugars, i.e., D-fructose (.433-fold), D-galactose (.171-fold), and D-glucose (.229-fold), was induced under conditions of N deficiency in this investigation. Similarly, it was reported that glucose and fructose accumulated under low-N conditions in *Synechocystis* (Asayama et al., 2004). In contrast, fructose and galactose did not vary markedly under high-light conditions in tomatoes, whereas they accumulated under low-light conditions (Urbanczyk-Wochniak and Fernie, 2005). Glucose and fructose are signaling molecules and energy sources in plants under conditions of abiotic stress (Bonneau et al., 2013). Although foliar starch, glucose, and fructose accumulate under low-N conditions, N deficiency inhibits photosynthetic capacity and growth. Poplar roots actively forage for nutrients under low-N conditions, while the acquired N appears insufficient for the biosynthesis of photosynthetic enzymes and metabolic precursors, leading to decreased photosynthetic capacity (Sardans and Peñuelas, 2012). In this study, the level of phytol related to chlorophyll synthesis increased significantly, which would directly affect the chlorophyll content in photosynthesis. Moreover, there was a marked accumulation of oxalic acid, which is involved in in the photosynthetic C3 cycle and photorespiration. Accumulation of phosphoric acid was also observed; this is involved in cyclic photophosphorylation. Interestingly, in contrast to most previous studies, four amines (putrescine, ethanolamine, ethylamine, and cadaverine) showed significant changes in this study. As components of N assimilation, the synthesis of ethylamine, ethanolamine, and putrescine decreased. Consistently, previous studies have shown that polyamines play an important role in modulating the sensitivity/tolerance to N stress (Syed et al., 2011). In addition to carbohydrate metabolic processes, N deficiency also affects the metabolism of other nutrients, such as phosphate and sulfur transport, indicating alteration of the N:C/P/S ratios and potential interactions (Supplementary Table S7). Specifically, several sugar-related pathways are known to interconnect with N-responsive pathways, and C/N balance has particular significance for plant growth. Obviously, crosstalk between N and C/P/S metabolism is involved in nutrient utilization and signal transduction, and a limitation of one element could cause imbalances in the nutrient network and affect the uptake, accumulation, and utilization of other nutrients (Schachtman and Shin, 2007; Rennenberg et al., 2010).

## Conclusion

In summary, this study revealed the global changes of transcripts and metabolites occurring in response to N deprivation in *P. tomentosa*. Specifically, 2,662 DEGs, 30 significantly changed metabolites, and three altered hormones were detected in our study. Combining transcriptomic and metabolic profiles, our investigation revealed a general depression of molecular and physiological metabolism under low N stress, including N absorption and assimilation, photosynthesis, glycolysis, and the TCA cycle. This extends our understanding of the relationships between responsive genes and downstream metabolic responses and provides a basis for the integrated and comprehensive analysis of molecular responses to N deficiency, which may improve understanding of the molecular and metabolic mechanisms underlying the phenotype of *P. tomentosa*, and the interaction of C and N under conditions of N deficiency.

## Data Availability Statement

The original contributions presented in the study are publicly available. This data can be found here: National Center for Biotechnology Information (NCBI) BioProject database under accession number PRJNA296440.

## Author Contributions

YW designed the experiment and edited the manuscript. MC and YY performed the research and wrote the manuscript. XY, LZ, TF, and XH contributed to the analytical tools and reagents. All the authors read and approved the final manuscript.

## Conflict of Interest

The authors declare that the research was conducted in the absence of any commercial or financial relationships that could be construed as a potential conflict of interest.

## Publisher’s Note

All claims expressed in this article are solely those of the authors and do not necessarily represent those of their affiliated organizations, or those of the publisher, the editors and the reviewers. Any product that may be evaluated in this article, or claim that may be made by its manufacturer, is not guaranteed or endorsed by the publisher.

## Footnotes

1
http://www.nist.gov/index.html

2
http://www.genome.jp/kegg/

3
http://www.umetrics.com/simca

4
http://www.bioconductor.org/packages/2.4/bioc/html/KEGGSOAP.html

5
http://www.cytoscape.org

6
http://trinityrnaseq.sourceforge.net/

7
http://www.ncbi.nlm.nih.gov

8
http://www.expasy.ch/sprot

9
http://www.ncbi.nlm.nih.gov/COG

10
http://blast.ncbi.nlm.nih.gov/Blast.cgi

11
http://www.blast2go.com/b2ghome

## References

[B1] Albinsky D. Kusano M. Higuchi M. Hayashi N. Kobayashi M. Fukushima A. (2010). Metabolomic screening applied to rice FOX *Arabidopsis* lines leads to the identification of a gene-changing nitrogen metabolism. *Mol. Plant* 3 125–142. 10.1093/mp/ssp069 20085895

[B2] Asayama M. Imamura S. Yoshihara S. Miyazaki A. Yoshida N. Sazuka T. (2004). SigC, the Group 2 sigma factor of RNA polymerase, contributes to the late-stage gene expression and nitrogen promoter recognition in the *Cyanobacterium Synechocystis* sp. Strain PCC 6803. *Biosci. Biotech. Biochem.* 68 477–487. 10.1271/bbb.68.477 15056876

[B3] Bai L. Deng H. Zhang X. Yu X. Li Y. (2016). Gibberellin is involved in inhibition of cucumber growth and nitrogen uptake at suboptimal root-zone temperatures. *PLoS One* 11:e0156188. 10.1371/journal.pone.0156188 27213554 PMC4877016

[B4] Bi Y.-M. Wang R.-L. Zhu T. Rothstein S. J. (2007). Global transcription profiling reveals differential responses to chronic nitrogen stress and putative nitrogen regulatory components in *Arabidopsis*. *BMC Genomics* 8:281. 10.1186/1471-2164-8-281 17705847 PMC1994689

[B5] Bonneau L. Huguet S. Wipf D. Pauly N. Truong H. N. (2013). Combined phosphate and nitrogen limitation generates a nutrient stress transcriptome favorable for arbuscular mycorrhizal symbiosis in *Medicago truncatula*. *New Phytol.* 199 188–202. 10.1111/nph.12234 23506613

[B6] Bot J. L. Bénard C. Robin C. Bourgaud F. Adamowicz S. (2009). The ‘trade-off’ between synthesis of primary and secondary compounds in young tomato leaves is altered by nitrate nutrition: experimental evidence and model consistency. *J. Exp. Bot.* 60 4301–4314. 10.1093/jxb/erp271 19741002

[B7] Bowsher C. G. Emes M. J. Cammack R. Hucklesby D. P. (1988). Purification and properties of nitrite reductase from roots of pea (*Pisum sativum* cv. Meteor). *Planta* 175 334–340. 10.1007/BF00396338 24221870

[B8] Broyart C. Fontaine J. X. Molinié R. Cailleu D. Tercé-Laforgue T. Dubois F. (2010). Metabolic profiling of maize mutants deficient for two glutamine synthetase isoenzymes using 1H-NMR-based metabolomics. *Phytochem. Anal.* 21 102–109. 10.1002/pca.1177 19866455

[B9] Cai H. Lu Y. Xie W. Zhu T. Lian X. (2012). Transcriptome response to nitrogen starvation in rice. *J. Biosci.* 37 731–747. 10.1007/s12038-012-9242-2 22922198

[B10] Cao Y. W. Qu R. J. Miao Y. J. Tang X. Q. Zhou Y. Wang L. (2019). Untargeted liquid chromatography coupled with mass spectrometry reveals metabolic changes in nitrogen-deficient Isatis indigotica Fortune. *Phytochemistry* 166 112058. 10.1016/j.phytochem.2019.112058 31280093 PMC7111722

[B11] Chen M. Bao H. Wu Q. Wang Y. (2015). Transcriptome-wide identification of mirna targets under nitrogen deficiency in Populus tomentosa using degradome sequencing. *Int. J. Mol. Sci.* 16 13937–13958. 10.3390/ijms160613937 26096002 PMC4490532

[B12] Chen M. Wang C. Bao H. Chen H. Wang Y. (2016). Genome-wide identification and characterization of novel lncRNAs in Populus under nitrogen deficiency. *Mol. Genet. Genomics* 291 1663–1680. 10.1007/s00438-016-1210-3 27138920

[B13] Chen Q. Liu Z. Wang B. Wang X. Lai J. Tian F. (2015). Transcriptome sequencing reveals the roles of transcription factors in modulating genotype by nitrogen interaction in maize. *Plant Cell Rep.* 34 1761–1771. 10.1007/s00299-015-1822-9 26116219 PMC4569664

[B14] Chen X. Zhu W. Azam S. Li H. Zhu F. Li H. (2013). Deep sequencing analysis of the transcriptomes of peanut aerial and subterranean young pods identifies candidate genes related to early embryo abortion. *Plant Biotechnol. J.* 11 115–127. 10.1111/pbi.12018 23130888

[B15] Chen Z.-H. Bao M.-L. Sun Y.-Z. Yang Y.-J. Xu X.-H. Wang J.-H. (2011). Regulation of auxin response by miR393-targeted transport inhibitor response protein1 is involved in normal development in *Arabidopsis*. *Plant Mol. Biol.* 77 619–629. 10.1007/s11103-011-9838-1 22042293

[B16] Dash M. Yordanov Y. S. Georgieva T. Kumari S. Wei H. Busov V. (2015). A systems biology approach identifies new regulators of poplar root development under low nitrogen. *Plant J.* 84 335–346. 10.1111/tpj.13002 26315649

[B17] de Bang T. C. Husted S. Laursen K. H. Persson D. P. Schjoerring J. K. (2021). The molecular-physiological functions of mineral macronutrients and their consequences for deficiency symptoms in plants. *New Phytol.* 229 2446–2469. 10.1111/nph.17074 33175410

[B18] Du Q. Wang B. Wei Z. Zhang D. Li B. (2012). Genetic diversity and population structure of Chinese White poplar (*Populus tomentosa*) revealed by SSR markers. *J. Hered.* 103 853–862. 10.1093/jhered/ess061 23008443

[B19] Fang L. Sun D. Xu Z. He J. Qi S. Chen X. (2015). Transcriptomic analysis of a moderately growing subisolate *Botryococcus braunii* 779 (Chlorophyta) in response to nitrogen deprivation. *Biotechnol. Biofuels* 8:130. 10.1186/s13068-015-0307-y 26322124 PMC4552190

[B20] Foyer C. H. Mullineaux P. M. (1998). The presence of dehydroascorbate and dehydroascorbate reductase in plant tissues. *FEBS Lett.* 425 528–529. 9563527 10.1016/s0014-5793(98)00281-6

[B21] Goel P. Bhuria M. Kaushal M. Singh A. K. (2016). Carbon: nitrogen interaction regulates expression of genes involved in N-uptake and assimilation in *Brassica juncea* L. *PLoS One* 11:e0163061. 10.1371/journal.pone.0163061 27637072 PMC5026376

[B22] Günther T. Lampei C. Schmid K. J. (2012). Mutational bias and gene conversion affect the intraspecific nitrogen stoichiometry of the *Arabidopsis thaliana* transcriptome. *Mol. Biol. Evol.* 30 561–568. 10.1093/molbev/mss249 23115321

[B23] Gutiérrez R. A. Lejay L. V. Dean A. Chiaromonte F. Shasha D. E. Coruzzi G. M. (2007). Qualitative network models and genome-wide expression data define carbon/nitrogen-responsive molecular machines in *Arabidopsis*. *Genome Biol.* 8 R7. 10.1186/gb-2007-8-1-r7 17217541 PMC1839130

[B24] Gutieìrrez R. A. Shasha D. E. Coruzzi G. M. (2005). Systems biology for the virtual plant. *Plant Physiol.* 138 550–554. 10.1104/pp.104.900150 15955912 PMC1150368

[B25] Hou J. Wu Q. Zuo T. Guo L. Chang J. Chen J. (2016). Genome-wide transcriptomic profiles reveal multiple regulatory responses of poplar to *Lonsdalea quercina* infection. *Trees* 30 1389–1402.

[B26] Ikram S. Bedu M. Daniel-Vedele F. Chaillou S. Chardon F. (2012). Natural variation of *Arabidopsis* response to nitrogen availability. *J. Exp. Bot.* 63 91–105. 21914659 10.1093/jxb/err244

[B27] Jackson L. E. Burger M. Cavagnaro T. R. (2008). Roots, nitrogen transformations, and ecosystem services. *Annu. Rev. Plant Biol.* 59 341–363. 10.1146/annurev.arplant.59.032607.092932 18444903

[B28] Jones D. L. Healey J. R. Willett V. B. Farrar J. F. Hodge A. (2005). Dissolved organic nitrogen uptake by plants—an important N uptake pathway? *Soil Biol. Biochem.* 37 413–423. 10.1016/j.soilbio.2004.08.008

[B29] Kamada-Nobusada T. Makita N. Kojima M. Sakakibara H. (2013). Nitrogen-dependent regulation of de novo cytokinin biosynthesis in rice: the role of glutamine metabolism as an additional signal. *Plant Cell Physiol.* 54 1881–1893. 10.1093/pcp/pct127 24058148 PMC3814184

[B30] Karp A. Shield I. (2008). Bioenergy from plants and the sustainable yield challenge. *New Phytol.* 179 15–32. 10.1111/j.1469-8137.2008.02432.x 18422906

[B31] Kiba T. Kudo T. Kojima M. Sakakibara H. (2010). Hormonal control of nitrogen acquisition: roles of auxin, abscisic acid, and cytokinin. *J. Exp. Bot.* 62 1399–1409. 10.1093/jxb/erq410 21196475

[B32] Kusano M. Fukushima A. Redestig H. Saito K. (2011). Metabolomic approaches toward understanding nitrogen metabolism in plants. *J. Exp. Bot.* 62 1439–1453. 21220784 10.1093/jxb/erq417

[B33] Liu Q. Chen X. Wu K. Fu X. (2015). Nitrogen signaling and use efficiency in plants: what’s new? *Curr. Opin. Plant Biol.* 27 192–198. 10.1016/j.pbi.2015.08.002 26340108

[B34] Liu W. Sun Q. Wang K. Du Q. Li W.-X. (2017). Nitrogen limitation adaptation (NLA) is involved in source-to-sink remobilization of nitrate by mediating the degradation of NRT1.7 in *Arabidopsis*. *New Phytol.* 214 734–744. 10.1111/nph.14396 28032637

[B35] Livak K. J. Schmittgen T. D. (2001). Analysis of relative gene expression data using real-time quantitative PCR and the 2(-Delta Delta C(T)) Method. *Methods* 25 402–408. 10.1006/meth.2001.1262 11846609

[B36] Lu Y. Sasaki Y. Li X. Mori I. C. Matsuura T. Hirayama T. (2015). ABI1 regulates carbon/nitrogen-nutrient signal transduction independent of ABA biosynthesis and canonical ABA signalling pathways in *Arabidopsis*. *J. Exp. Bot.* 66 2763–2771. 10.1093/jxb/erv086 25795738 PMC4986877

[B37] Luo J. Li H. Liu T. Polle A. Peng C. Luo Z.-B. (2013). Nitrogen metabolism of two contrasting poplar species during acclimation to limiting nitrogen availability. *J. Exp. Bot.* 64 4207–4224. 10.1093/jxb/ert234 23963674 PMC3808312

[B38] Luo J. Zhou J. Li H. Shi W. Polle A. Lu M. (2015). Global poplar root and leaf transcriptomes reveal links between growth and stress responses under nitrogen starvation and excess. *Tree Physiol.* 35 1283–1302. 10.1093/treephys/tpv091 26420789

[B39] Mesnard F. Ratcliffe R. G. (2005). NMR analysis of plant nitrogen metabolism. *Photosynth. Res.* 83 163–180. 10.1007/s11120-004-2081-8 16143850

[B40] Mi G. Chen F. Zhang F. (2008). Multiple signaling pathways controls nitrogen-mediated root elongation in maize. *Plant Signal. Behav.* 3 1030–1032. 10.4161/psb.6800 19704443 PMC2633766

[B41] Mockaitis K. Estelle M. (2008). Auxin receptors and plant development: a new signaling paradigm. *Annu. Rev. Cell Dev. Biol.* 24 55–80. 10.1146/annurev.cellbio.23.090506.123214 18631113

[B42] Mu X. Chen Y. (2021). The physiological response of photosynthesis to nitrogen deficiency. *Plant Physiol. Biochem.* 158 76–82. 10.1016/j.plaphy.2020.11.019 33296848

[B43] Murashige T. Skoog F. (1962). A revised medium for rapid growth and bio assays with tobacco tissue cultures. *Physiol. Plant.* 15 473–497. 10.1111/j.1399-3054.1962.tb08052.x

[B44] North K. A. Ehlting B. Koprivova A. Rennenberg H. Kopriva S. (2009). Natural variation in *Arabidopsis* adaptation to growth at low nitrogen conditions. *Plant Physiol. Biochem.* 47 912–918. 10.1016/j.plaphy.2009.06.009 19628403

[B45] Nunes-Nesi A. Fernie A. R. Stitt M. (2010). Metabolic and signaling aspects underpinning the regulation of plant carbon nitrogen interactions. *Mol. Plant* 3 973–996. 10.1093/mp/ssq049 20926550

[B46] Pan C. Valente J. J. LoBrutto R. Pickett J. S. Motto M. (2010). Combined application of high resolution and tandem mass spectrometers to characterize methionine oxidation in a parathyroid hormone formulation. *J. Pharm. Sci.* 99 1169–1179. 10.1002/jps.21901 19711445

[B47] Patterson K. Cakmak T. Cooper A. Lager I. Rasmusson A. G. Escobar M. A. (2010). Distinct signalling pathways and transcriptome response signatures differentiate ammonium- and nitrate-supplied plants. *Plant Cell Environ.* 33 1486–1501. 10.1111/j.1365-3040.2010.02158.x 20444219 PMC2920365

[B48] Rejeb I. B. Pastor V. Mauch-Mani B. (2014). Plant responses to simultaneous biotic and abiotic stress: molecular mechanisms. *Plants (Basel)* 3 458–475. 10.3390/plants3040458 27135514 PMC4844285

[B49] Ren Y. Sun F. Hou J. Chen L. Zhang Y. Kang X. (2015). Differential profiling analysis of miRNAs reveals a regulatory role in low N stress response of Populus. *Funct. Integr. Genomics* 15 93–105. 10.1007/s10142-014-0408-x 25398555

[B50] Rennenberg H. Wildhagen H. Ehlting B. (2010). Nitrogen nutrition of poplar trees. *Plant Biol. (Stuttg)* 12 275–291. 10.1111/j.1438-8677.2009.00309.x 20398235

[B51] Rhoades M. W. Reinhart B. J. Lim L. P. Burge C. B. Bartel B. Bartel D. P. (2002). Prediction of plant microRNA targets. *Cell* 110 513–520. 12202040 10.1016/s0092-8674(02)00863-2

[B52] Sakakibara H. (2003). Nitrate-specific and cytokinin-mediated nitrogen signaling pathways in plants. *J. Plant Res.* 116 253–257. 10.1007/s10265-003-0097-3 12836045

[B53] Sardans J. Peñuelas J. (2012). The role of plants in the effects of global change on nutrient availability and stoichiometry in the plant-soil system. *Plant Physiol.* 160 1741–1761. 10.1104/pp.112.208785 23115250 PMC3510107

[B54] Schachtman D. P. Shin R. (2007). Nutrient sensing and signaling: NPKS. *Annu. Rev. Plant Biol.* 58 47–69. 10.1146/annurev.arplant.58.032806.103750 17067284

[B55] Scheible W. R. Gonzalez-Fontes A. Lauerer M. Muller-Rober B. Caboche M. Stitt M. (1997). Nitrate acts as a signal to induce organic acid metabolism and repress starch metabolism in tobacco. *Plant Cell* 9 783–798. 10.1105/tpc.9.5.783 12237366 PMC156956

[B56] Schlüter U. Mascher M. Colmsee C. Scholz U. Bräutigam A. Fahnenstich H. (2012). Maize source leaf adaptation to nitrogen deficiency affects not only nitrogen and carbon metabolism but also control of phosphate homeostasis. *Plant Physiol.* 160 1384–1406. 10.1104/pp.112.204420 22972706 PMC3490595

[B57] Shao C. H. Qiu C. F. Qian Y. F. Liu G. R. (2020). Nitrate deficiency decreased photosynthesis and oxidation-reduction processes, but increased cellular transport, lignin biosynthesis and flavonoid metabolism revealed by RNA-Seq in Oryza sativa leaves. *PLoS One* 15:e0235975. 10.1371/journal.pone.0235975 32649704 PMC7351185

[B58] Shi H. W. Wang L. Y. Li X. X. Liu X. M. Hao T. Y. He X. J. (2016). Genome-wide transcriptome profiling of nitrogen fixation in *Paenibacillus* sp. WLY78. *BMC Microbiol.* 16:25. 10.1186/s12866-016-0642-6 26931570 PMC4774088

[B59] Sinha S. K. Rani M. Bansal N. Gayatri, Venkatesh K. Mandal P. K. (2015). Nitrate starvation induced changes in root system architecture, carbon:nitrogen metabolism, and mirna expression in nitrogen-responsive wheat genotypes. *Appl. Biochem. Biotechnol.* 177 1299–1312. 10.1007/s12010-015-1815-8 26315134

[B60] Studer M. H. DeMartini J. D. Davis M. F. Sykes R. W. Davison B. Keller M. (2011). Lignin content in natural *Populus* variants affects sugar release. *Proc. Natl. Acad. Sci. U.S.A.* 108 6300–6305. 10.1073/pnas.1009252108 21444820 PMC3076829

[B61] Suárez M. F. Avila C. Gallardo F. Cantón F. R. García-Gutiérrez A. Claros M. G. (2002). Molecular and enzymatic analysis of ammonium assimilation in woody plants. *J. Exp. Bot.* 53 891–904. 10.1093/jexbot/53.370.891 11912232

[B62] Syed D. N. Afaq F. Maddodi N. Johnson J. J. Sarfaraz S. Ahmad A. (2011). Inhibition of human melanoma cell growth by the dietary flavonoid fisetin is associated with disruption of Wnt/β-catenin signaling and decreased Mitf levels. *J. Invest. Dermatol.* 131 1291–1299. 10.1038/jid.2011.6 21346776 PMC3166244

[B63] Takei K. Ueda N. Aoki K. Kuromori T. Hirayama T. Shinozaki K. (2004). AtIPT3 is a Key Determinant of nitrate-dependent cytokinin biosynthesis in *Arabidopsis*. *Plant Cell Physiol.* 45 1053–1062. 10.1093/pcp/pch119 15356331

[B64] Tian J. Chen J. Li B. Zhang D. (2016). Association genetics in Populus reveals the interactions between Pto-miR160a and its target Pto-ARF16. *Mol. Genet. Genomics* 291 1069–1082. 10.1007/s00438-015-1165-9 26732268

[B65] Trachsel S. Kaeppler S. M. Brown K. M. Lynch J. P. (2013). Maize root growth angles become steeper under low N conditions. *Field Crops Res.* 140 18–31. 10.1016/j.fcr.2012.09.010

[B66] Tschoep H. Gibon Y. Carillo P. Armengaud P. Szecowka M. Nunes-Nesi A. (2009). Adjustment of growth and central metabolism to a mild but sustained nitrogen-limitation in *Arabidopsis*. *Plant Cell Environ.* 32 300–318. 10.1111/j.1365-3040.2008.01921.x 19054347

[B67] Tuskan G. A. Groover A. T. Schmutz J. DiFazio S. P. Myburg A. Grattapaglia D. (2018). Hardwood tree genomics: unlocking woody plant biology. *Front. Plant Sci.* 9:1799. 10.3389/fpls.2018.01799 30619389 PMC6304363

[B68] Urbanczyk-Wochniak E. Fernie A. R. (2005). Metabolic profiling reveals altered nitrogen nutrient regimes have diverse effects on the metabolism of hydroponically-grown tomato (*Solanum lycopersicum*) plants. *J. Exp. Bot.* 56 309–321. 10.1093/jxb/eri059 15596475

[B69] Vega A. Canessa P. Hoppe G. Retamal I. Moyano T. C. Canales J. (2015). Transcriptome analysis reveals regulatory networks underlying differential susceptibility to Botrytis cinerea in response to nitrogen availability in *Solanum lycopersicum*. *Front. Plant Sci.* 6:911. 10.3389/fpls.2015.00911 26583019 PMC4631835

[B70] Vidal E. A. Araus V. Lu C. Parry G. Green P. J. Coruzzi G. M. (2010). Nitrate-responsive miR393/AFB3 regulatory module controls root system architecture in *Arabidopsis thaliana*. *Proc. Natl. Acad. Sci. U.S.A.* 107 4477–4482. 10.1073/pnas.0909571107 20142497 PMC2840086

[B71] Vogel C. Bodenhausen N. Gruissem W. Vorholt J. A. (2016). The *Arabidopsis* leaf transcriptome reveals distinct but also overlapping responses to colonization by phyllosphere commensals and pathogen infection with impact on plant health. *New Phytol.* 212 192–207. 10.1111/nph.14036 27306148

[B72] Wang J. Ma X.-M. Kojima M. Sakakibara H. Hou B.-K. (2013). Glucosyltransferase UGT76C1 finely modulates cytokinin responses via cytokinin N-glucosylation in *Arabidopsis thaliana*. *Plant Physiol. Biochem.* 65 9–16. 10.1016/j.plaphy.2013.01.012 23416491

[B73] Wang X. Li X. Zhang S. Korpelainen H. Li C. (2016). Physiological and transcriptional responses of two contrasting *Populus* clones to nitrogen stress. *Tree Physio.l* 36 628–642. 10.1093/treephys/tpw019 27095258 PMC4886292

[B74] Wang Y. N. Tang L. Hou Y. Wang P. Yang H. Wei C. L. (2016). Differential transcriptome analysis of leaves of tea plant (*Camellia sinensis*) provides comprehensive insights into the defense responses to Ectropis oblique attack using RNA-Seq. *Funct. Integr. Genomics* 16 383–398. 10.1007/s10142-016-0491-2 27098524

[B75] Wei H. Yordanov Y. Georgieva T. Li X. Busov V. (2013a). Nitrogen deprivation promotes *Populus* root growth through global transcriptome reprogramming and activation of hierarchical genetic networks. *New Phytol.* 200 483–497. 10.1111/nph.12375 23795675

[B76] Wei H. Yordanov Y. Kumari S. Georgieva T. Busov V. (2013b). Genetic networks involved in poplar root response to low nitrogen. *Plant Signal. Behav.* 8 e27211. 10.4161/psb.27211 24300216 PMC4091351

[B77] Woo H. Koo H. Kim J. Jeong H. Yang J. O. Lee I. (2016). Programming of plant leaf senescence with temporal and inter-organellar coordination of transcriptome in *Arabidopsis*. *Plant Physiol.* 171 452–467. 10.1104/pp.15.01929 26966169 PMC4854694

[B78] Yang J. H. Han S. J. Yoon E. K. Lee W. S. (2006). Evidence of an auxin signal pathway, microRNA167-ARF8-GH3, and its response to exogenous auxin in cultured rice cells. *Nucleic Acids Res.* 34 1892–1899. 10.1093/nar/gkl118 16598073 PMC1447648

[B79] Yang W. Yoon J. Choi H. Fan Y. Chen R. An G. (2015). Transcriptome analysis of nitrogen-starvation-responsive genes in rice. *BMC Plant Biol.* 15:31. 10.1186/s12870-015-0425-5 25644226 PMC4333837

[B80] Yu J. Han J. Wang R. Li X. (2016). Down-regulation of nitrogen/carbon metabolism coupled with coordinative hormone modulation contributes to developmental inhibition of the maize ear under nitrogen limitation. *Planta* 244 111–124. 10.1007/s00425-016-2499-1 26979324

[B81] Zahran H. H. (1999). Rhizobium-legume symbiosis and nitrogen fixation under severe conditions and in an arid climate. *Microbiol. Mol. Biol. Rev.* 63 968–989. 10.1128/MMBR.63.4.968-989.1999 10585971 PMC98982

[B82] Zhang J. Liang S. Duan J. Wang J. Chen S. Cheng Z. (2012). De novo assembly and characterisation of the transcriptome during seed development, and generation of genic-SSR markers in Peanut (*Arachis hypogaea* L.). *BMC Genomics* 13:90. 10.1186/1471-2164-13-90 22409576 PMC3350410

[B83] Zhao W. Yang X. Yu H. Jiang W. Sun N. Liu X. (2015). RNA-Seq-based transcriptome profiling of early nitrogen deficiency response in cucumber seedlings provides new insight into the putative nitrogen regulatory network. *Plant Cell Physiol.* 56 455–467. 10.1093/pcp/pcu172 25432971

[B84] Zheng Z. L. (2009). Carbon and nitrogen nutrient balance signaling in plants. *Plant Signal. Behav.* 4 584–591. 10.4161/psb.4.7.8540 19820356 PMC2710548

