# Supplementary material for: Metabolomics and Transcriptomics Integration of Early Response of Populus tomentosa to Reduced Nitrogen Availability
Source: Front Plant Sci. 2021 Dec 8;12:769748. doi: 10.3389/fpls.2021.769748 (PMC8692568; doi:10.3389/fpls.2021.769748)
Supplement: Supplementary file 1 [file Data_Sheet_1.DOCX]

**Supplementary Figure S1**. Pipeline of bioinformatics analysis for transcriptome in *Populus tomentosa*.


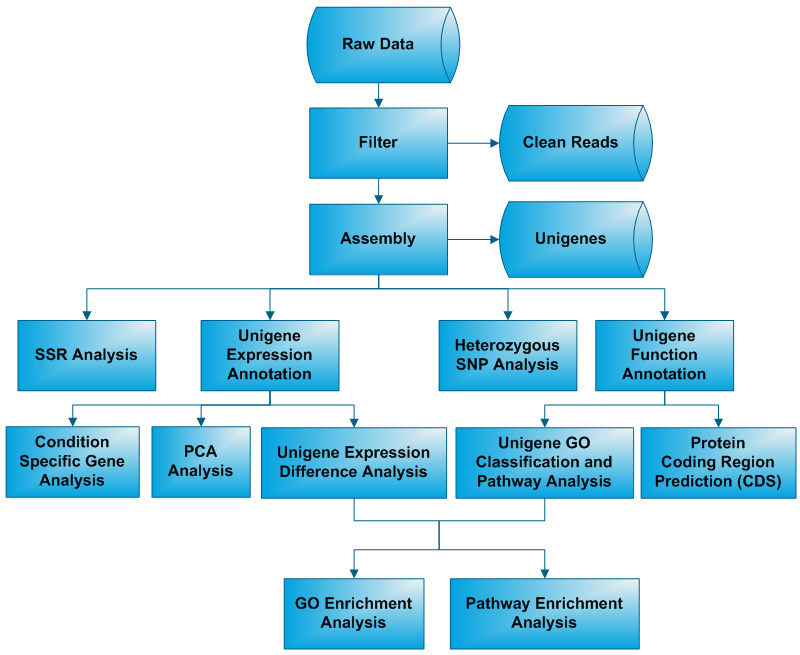


Note: Raw data were filtered to remove adaptors and low-quality reads to obtain clean reads.Then clean reads were processed to transcriptome *de novo* assembly using Trinity, and the length distribution of Contigs and Unigenes was calculated. Four parts of analysis of unigenes were done: SSR analysis, SNP analysis, unigene expression annotation and unigene function annotation including blast against with databases of NR, NT, Swiss-Prot, KEGG, COG and GO.
