# Supplementary material for: Metabolomics and Transcriptomics Integration of Early Response of Populus tomentosa to Reduced Nitrogen Availability
Source: Front Plant Sci. 2021 Dec 8;12:769748. doi: 10.3389/fpls.2021.769748 (PMC8692568; doi:10.3389/fpls.2021.769748)

**Supplementary Figure S2.** The total ion chromatogram (TIC) of *Populus tomentosa with*  sufficient N condition (KK) or low N condition (DN).

A

DN1


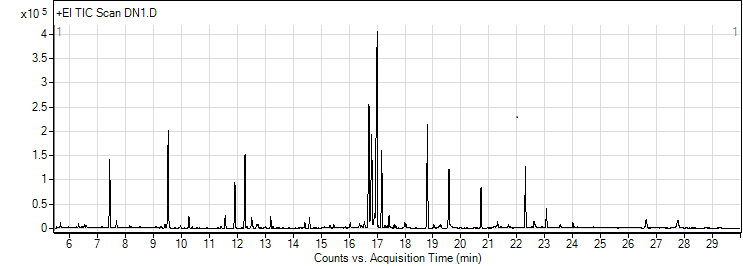


B

DN2

C

DN3


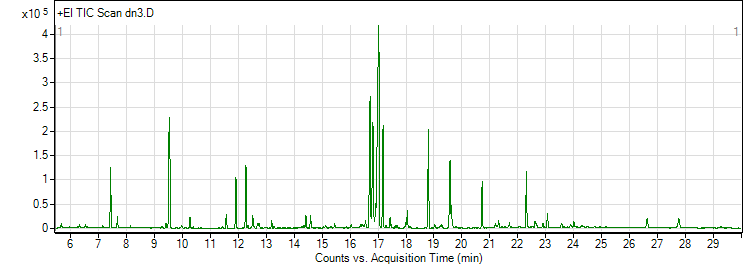


D

DN4


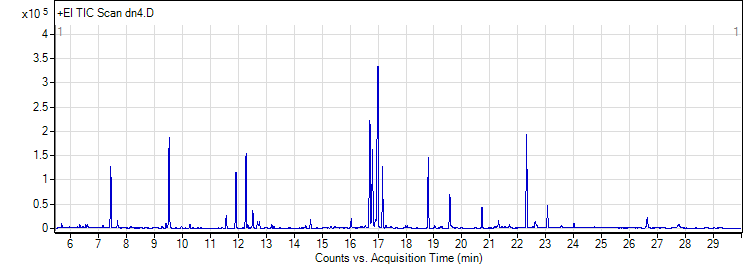


E

DN5


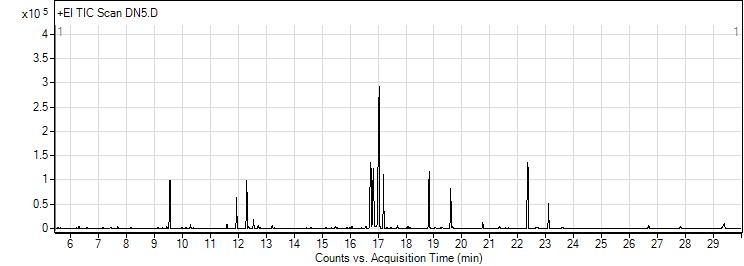


F

DN6


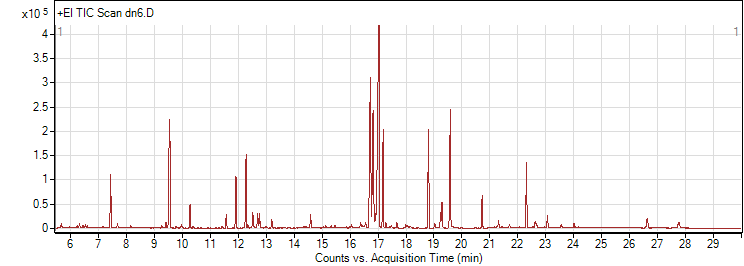


H

KK1


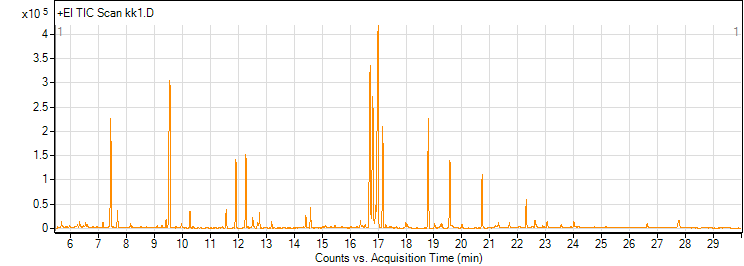


I

KK2


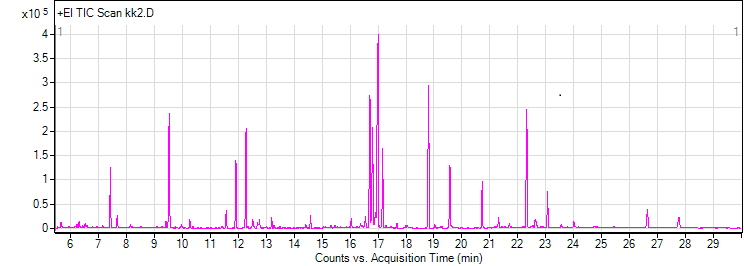


J

KK3


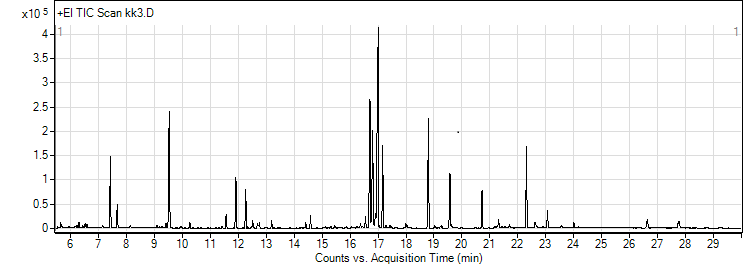


K

KK4


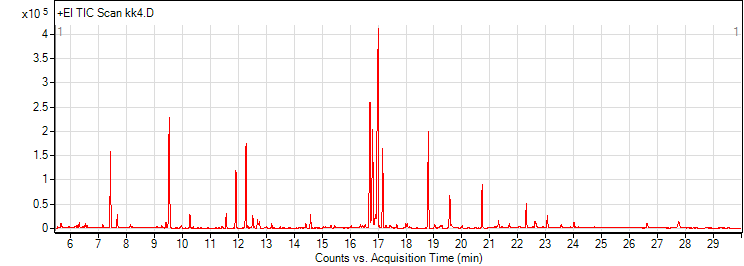


L

KK5


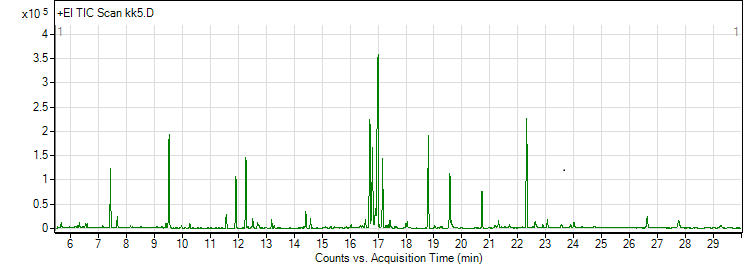


M

KK6


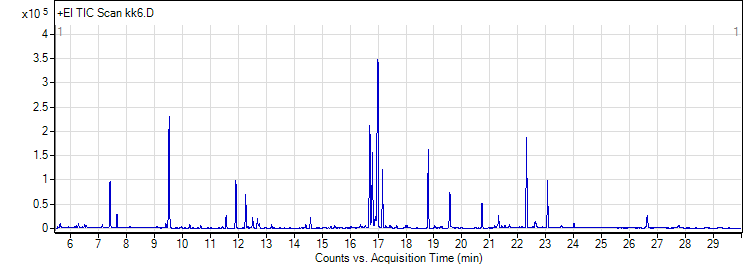

Supplement: Supplementary file 2 [file Data_Sheet_2.DOC]
