# Supplementary material for: Metabolomics and Transcriptomics Integration of Early Response of Populus tomentosa to Reduced Nitrogen Availability
Source: Front Plant Sci. 2021 Dec 8;12:769748. doi: 10.3389/fpls.2021.769748 (PMC8692568; doi:10.3389/fpls.2021.769748)
Supplement: Supplementary file 3 [file Data_Sheet_3.DOC]

**Supplementary Figure S3.**  Partial least squares-discriminant analysis (PLS-DA) and principal component analysis (PCA) of *Populus tomentosa with*  sufficient N condition(KK) or low N treatment (DN).

A: Partial least squares-discriminant analysis (PLS-DA) analysis.

B: Principal component analysis (PCA) analysis.
