# Supplementary material for: Metabolomics and Transcriptomics Integration of Early Response of Populus tomentosa to Reduced Nitrogen Availability
Source: Front Plant Sci. 2021 Dec 8;12:769748. doi: 10.3389/fpls.2021.769748 (PMC8692568; doi:10.3389/fpls.2021.769748)
Supplement: Supplementary file 4 [file Data_Sheet_4.DOC]

#### Supplementary Figure S4. List of differentially expressed genes (FDR≤0.001 and |log_2_Ratio|≥1).


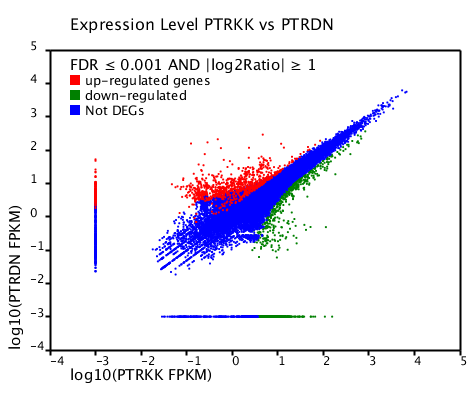


Notes: The genes were classified into three classes. Red genes are up-regulated that gene expression of right sample is larger than left sample. Green genes are down-regulated that gene expression of left sample is larger than right sample. Blue genes are not differentially expressed genes. The horizontal coordinates is the expression level of right and the vertical coordinates is the expression level of left sample

#### 
