# Supplementary material for: Metabolomics and Transcriptomics Integration of Early Response of Populus tomentosa to Reduced Nitrogen Availability
Source: Front Plant Sci. 2021 Dec 8;12:769748. doi: 10.3389/fpls.2021.769748 (PMC8692568; doi:10.3389/fpls.2021.769748)

#### **Supplementary Figure S5**. Comparison of genes expression correlation of *Populus tomentosa* between qRT-PCR and RNA-seq with a *p*-value of 0.01. Log*_2_* of fold changes are plotted for a handful of ten genes with differential expression in *P. tomentosa* treated or untreated with low nitrogen stress for three days.


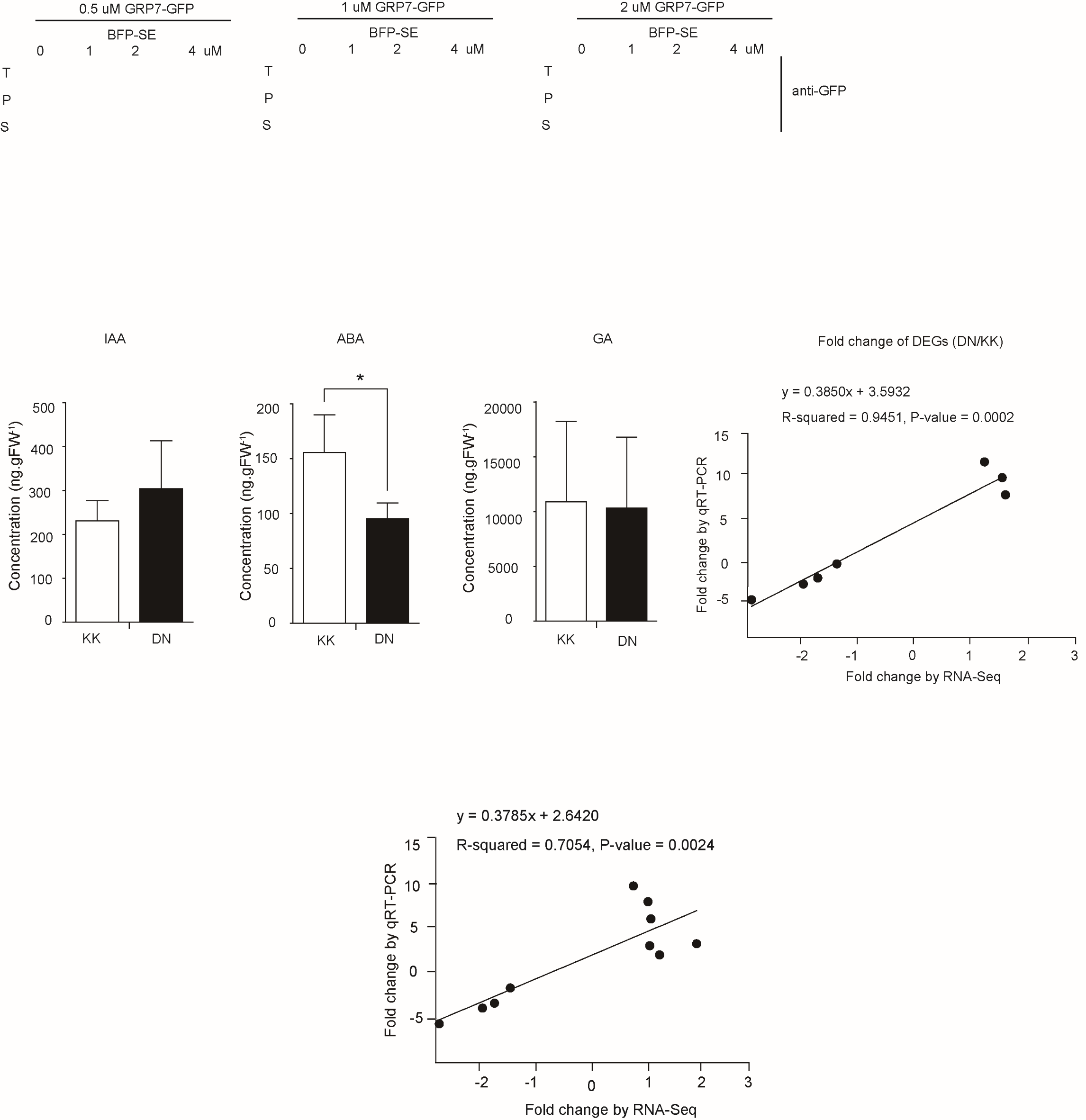

Supplement: Supplementary file 5 [file Data_Sheet_5.DOCX]
