# Supplementary material for: Metabolomics and Transcriptomics Integration of Early Response of Populus tomentosa to Reduced Nitrogen Availability
Source: Front Plant Sci. 2021 Dec 8;12:769748. doi: 10.3389/fpls.2021.769748 (PMC8692568; doi:10.3389/fpls.2021.769748)
Supplement: Supplementary file 6 [file Table_1.DOCX]

**Supplementary Table S1.** The pimers of 14 DEGs used for qRT-PCR in *Populus tomentosa*

under low N stress.

| **Primer** | **Sequence (5**'-**3**') |
| --- | --- |
| Unigene22453F | TGGGAGGGATCCTAAGATATGG |
| Unigene22453R | CAGCTCCGAAGGGCATTAG |
| Unigene19959F | GGGTCCATCATTGTCATCATTTAG |
| Unigene19959R | CATCAGAAGACGTCGGAAGAA |
| CL933.Contig1F | GAACGCGTCTTCGCCTAATA |
| CL933.Contig1R | CGGGACGAGAGCAACATTTA |
| Unigene24651F | CACGTCAAGAGCTAGAGAGAGA |
| Unigene24651R | CTGTGGATGGACACCGAAATAG |
| Unigene3952F | GTCGGTGATATTGTCTGGGTTA |
| Unigene3952R | GTCACTCTGGACAGCATACTT |
| CL5328.Contig5F | GTTGCCGGAGTCTTCTCTATG |
| CL5328.Contig5R | CCAACATCCTGGACCCTATTG |
| Unigene24078F | GATGATGATATCCCTGCAATTTATACC |
| Unigene24078R | GTTTGTGAAGCATTGGAGAGAAA |
| CL9290.Contig2F | TCCCTTTCGCTACAAATCTCC |
| CL9290.Contig2R | GGTGATGGAAGGGCTTATCTT |
| Unigene25149F | TGAAACTGGGTCTTAGCATTGA |
| Unigene25149R | TTAGTCCACCTCCTCCATCTT |
| Unigene13209F | TGCCACAAACCCGATCAA |
| Unigene13209R | CTTCACGAAGAACCCAACAAATC |
| Unigene18608F | ATGGCAGTAACATAGAACAGT |
| Unigene18608R | CAGTCTCCTCCAGGCAAA |
| Unigene3286F | GGTTCTTATGCGAGTATTG |
| Unigene3286R | TTGCGAAGATTTGCAGTCA |
| CL6853.Contig2F | GGGCAATTCCGATGTTTA |
| CL6853.Contig2R | GGTCGCACTCCATTAGGC |
| CL4139.Contig1F | TATGGCAAGGGAGGGAAGA |
| CL4139.Contig1R | ATGGCTTATGAACACCACAACT |
