# Supplementary material for: Metabolomics and Transcriptomics Integration of Early Response of Populus tomentosa to Reduced Nitrogen Availability
Source: Front Plant Sci. 2021 Dec 8;12:769748. doi: 10.3389/fpls.2021.769748 (PMC8692568; doi:10.3389/fpls.2021.769748)
Supplement: Supplementary file 9 [file Table_4.DOCX]

**Supplementary Table S4.** Output statistics of transcriptome sequencing.

| Samples | Total Raw Reads | Total Clean Reads | Total Clean Nucleotides (nt) | Q20 percentage | N percentage | GC percentage |
| --- | --- | --- | --- | --- | --- | --- |
| DN | 55,471,194 | 52,903,032 | 4,761,272,880 | 99.14% | 0.00% | 44.90% |
| KK | 54,650,854 | 51,940,444 | 4,674,639,960 | 99.16% | 0.00% | 45.05% |

**Notes**：Q20 percentage represents the proportion of nucleotides with quality value larger than 20; N percentage is proportion of unknown nucleotides in clean reads; GC percentage is proportion of guanidine and cytosine nucleotides among total nucleotides.
