# Supplementary material for: Metabolomics and Transcriptomics Integration of Early Response of Populus tomentosa to Reduced Nitrogen Availability
Source: Front Plant Sci. 2021 Dec 8;12:769748. doi: 10.3389/fpls.2021.769748 (PMC8692568; doi:10.3389/fpls.2021.769748)
Supplement: Supplementary file 10 [file Table_5.DOCX]

**Supplementary Table S5.** [Summary of assembly transcripts](file:///E:\2014-9-30%20低氮转录组测序全部数据-1\F13TSFNCKF0342_POPdmxT\assembly\assembly_statistic.xls) for *Populus tomentosa.*

|  | Sample | Total Number | Total Length(nt) | Mean Length (nt) | N50 | Total Consensus Sequences | Distinct Clusters | Distinct Singletons |
| --- | --- | --- | --- | --- | --- | --- | --- | --- |
| Contig | PTRDN | 154,857 | 44,212,165 | 286 | 406 | - | - | - |
|  | PTRKK | 146,823 | 43,161,905 | 294 | 392 | - | - | - |
| Unigene | PTRDN | 71,801 | 41,819,345 | 582 | 948 | 71,801 | 25,382 | 46,419 |
|  | PTRKK | 82,908 | 42,130,601 | 508 | 801 | 82,908 | 26,351 | 56,557 |
|  | All | 69,523 | 48,263,359 | 694 | 1088 | 69,523 | 26,953 | 42,570 |
