# Supplementary material for: Metabolomics and Transcriptomics Integration of Early Response of Populus tomentosa to Reduced Nitrogen Availability
Source: Front Plant Sci. 2021 Dec 8;12:769748. doi: 10.3389/fpls.2021.769748 (PMC8692568; doi:10.3389/fpls.2021.769748)
Supplement: Supplementary file 11 [file Table_6.DOCX]

**Supplementary Table S6.** [Annotation results](file:///E:\2014-9-30%20低氮转录组测序全部数据-1\F13TSFNCKF0342_POPdmxT\annotation\annotation_statistic.xls) of unigenes in *Populus tomentosa.*

| Sequence File | Number of annotated unigenes | Percentage of annotated unigene |
| --- | --- | --- |
| Total unigene | 59,125 | 100 |
| NR | 52,816 | 89.33% |
| NT | 54,584 | 92.32% |
| Swiss-Prot | 32,342 | 54.70% |
| KEGG | 29,222 | 49.42% |
| COG | 18,431 | 31.17% |
| GO | 42,264 | 71.48% |
| CDS | 51,666 | 87.38% |
